# (−)-Epicatechin-3-O-β-D-allopyranoside from *Davallia formosana* prevents diabetes and dyslipidemia in streptozotocin-induced diabetic mice

**DOI:** 10.1371/journal.pone.0173984

**Published:** 2017-03-23

**Authors:** Cheng-Hsiu Lin, Jin-Bin Wu, Jia-Ying Jian, Chun-Ching Shih

**Affiliations:** 1 Department of Internal Medicine, Fengyuan Hospital, Ministry of Health and Welfare, Fengyuan District, Taichung City, Taiwan; 2 Graduate Institute of Pharmaceutical Chemistry, China Medical University, Taichung City, Taiwan; 3 Graduate Institute of Biotechnology and Biomedical Engineering, College of Health Science, Central Taiwan University of Science and Technology, Taichung City, Taiwan; Stellenbosch University, SOUTH AFRICA

## Abstract

The objective of this study was to evaluate the effects and molecular mechanism of (−)-epicatechin-3-O-β-D-allopyranoside from *Davallia formosana* (BB) (also known as Gu-Sui-Bu) on type 1 diabetes mellitus and dyslipidemia in streptozotocin (STZ)-induced diabetic mice. This plant was demonstrated to display antioxidant activities and possess polyphenol contents. Diabetic mice were randomly divided into six groups and were given daily oral gavage doses of either BB (at three dosage levels), metformin (Metf) (at 0.3 g/kg body weight), fenofibrate (Feno) (at 0.25 g/kg body weight) or vehicle (distilled water) and a group of control (CON) mice were gavaged with vehicle over a period of 4 weeks. Treatment with BB led to reduced levels of blood glucose, HbA_1C_, triglycerides and leptin and to increased levels of insulin and adiponectin compared with the vehicle-treated STZ group. The diabetic islets showed retraction from their classic round-shaped as compared with the control islets. The BB-treated groups (at middle and high dosages) showed improvement in islets size and number of Langerhans islet cells. The membrane levels of skeletal muscular glucose transporter 4 (GLUT4) were significantly higher in BB-treated mice. This resulted in a net glucose lowering effect among BB-treated mice. Moreover, BB enhanced the expression of skeletal muscle phospho-AMPK in treated mice. BB-treated mice increased expression of fatty acid oxidation enzymes, including peroxisome proliferator-activated receptor α (PPARα) and mRNA levels of carnitine palmitoyl transferase Ia (CPT1a). These mice also expressed lower levels of lipogenic genes such as fatty acid synthase (FAS), as well as lower mRNA levels of sterol regulatory element binding protein 1c (SREBP1c) and liver adipocyte fatty acid binding protein 2 (aP2). This resulted in a reduction in plasma triglyceride levels. BB-treated mice also expressed lower levels of PPARγ and FAS protein. This led to reduced adipogenesis, fatty acid synthesis and lipid accumulation within adipose tissue, and consequently, to lower triglyceride levels in liver, blood, and adipose tissue. Moreover, BB treatment not only displayed the activation Akt in liver tissue and skeletal muscle, but also in C2C12 myotube to cause an increase in phosphorylation of Akt in the absence of insulin. These results demonstrated that BB act as an activator of AMPK and /or regulation of insulin pathway (Akt), and the antioxidant activity within the pancreas. Therefore, BB treatment ameliorated the diabetic and dyslipidemic state in STZ-induced diabetic mice.

## Introduction

Diabetes mellitus is an increasingly prevalent metabolic disorder, and it may be accompanied by hyperglycemia, hyperlipidemia, and various cardiovascular diseases. Diabetes mellitus type 1 is characterized by chronic hyperglycemia, through pathophysiology that involves destruction of pancreatic β-cells. Type 1 diabetes affects an estimated 5~10% of all diabetes patients [1].

Streptozotocin (STZ) is widely used to induce type 1 diabetes mellitus in experimental animal models [2]. STZ is a pancreatic β-cell toxin, and it can easily induce type 1 diabetes when administered in either a single large dose or in repeated low doses over several days [3]. The STZ-induced diabetic rodent model is usually characterized by hyperglycemia in both the fasting and non-fasting state, reduced serum insulin levels and hyperlipidemia [4]. STZ is a nitric oxide donor, nitric oxide was found to bring about the destruction of pancreatic islet cells and STZ by itself was found to generate reactive oxygen species, which contributed to DNA fragmentation and evoked other deleterious changes within the pancreatic tissue [5,6]. STZ-induction cause a production of reactive oxygen species within the pancreatic tissue, and thus it has been used to study natural compounds whether exhibit the antioxidant and protective activity of STZ-induced oxidative stress [7].

Gu-Sui-Bu [*Drynaria fortunei* (Kunze)] is a traditional Chinese medicine that is used to treat rheumatoid arthritis and bone disorders. *Davallia formosana* Hayata (Davalliaceae) is also known as Gu-Sui-Bu in the Taiwanese herbal market and the plant contains several bioactive compounds that include davallic acid [8], flavan-3-ol, and proanthocyanidin allosides [9].

**Fig 1:**
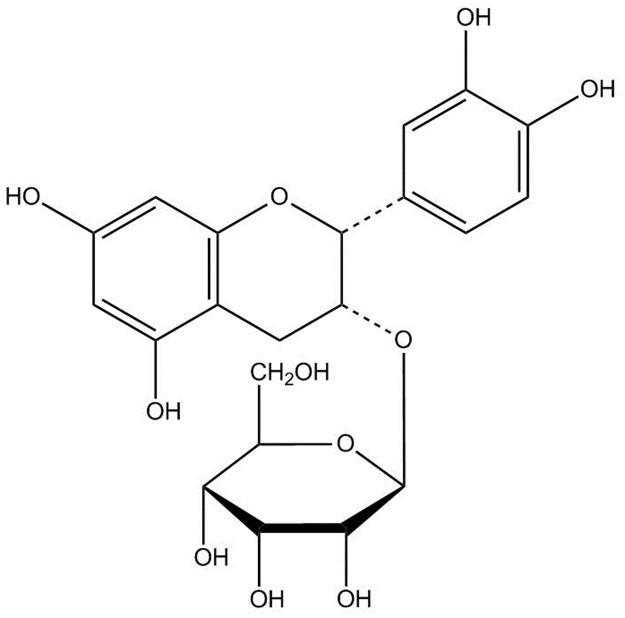
Structure of (−)-epicatechin-3-O-β-D-allopyranoside (BB).

Evidence suggests that many antidiabetic drugs have beneficial effects on osteogenesis. Use of (−)-epicatechin-3-O-β-D-allopyranoside (BB) (Fig 1) from *Davallia formosana* has mitigated bone loss. This plant was demonstrated to possess the antioxidant activity and have the ability to scavenge free radical radicals (including scavenging against DPPH radicals), and possess polyphenol contents [10]. Polyphenols are demonstrated to protect cell constituents against oxidative stress and reduce tissue damage by acting directly on reactive oxygen species or by stimulating endogenous antioxidant defense systems [11]. However, the antihyperglycemic and antihyperlipidemic effects of BB in STZ-induced mice remain unknown.

Glucose transporter type 4 (GLUT4) plays an essential role in blood glucose homeostasis. Skeletal muscle is the primary site of insulin-mediated glucose uptake in the body [12]. Increased fasting insulin levels promote GLUT4 translocation from intracellular storage sites to the plasma membrane in skeletal muscle [13,14]. In skeletal muscle, glucose transport is activated by insulin and by muscle contraction and/or exercise [15,16].

There are two principal mechanisms involved in promoting translocation of GLUT4 to the plasma membrane including insulin signaling through phosphatidylinositol 3’ kinase (PI3-kinase) /Akt pathway and the AMPK pathway [16].

Metformin reduces lipid levels through activation of hepatic and striated muscle AMP-activated protein kinase (AMPK) [17]. Fenofibrate is an agonist of peroxisome proliferator-activated receptor α (PPARα), in clinical use to treat high blood levels of lipids and triglyceride [18], and act by modulating several genes involved in lipogenesis and fatty acid oxidation and fenofibrate enhances femoral bone mineral density [18,19].

Evidence suggests that phosphorylation of Threonine 172 (Thr172) of the α subunit is essential for AMPK activity [20]. The current study was aimed to gain insight into whether BB can induce favorable metabolic activity, including glucose and lipid lowering effects, by regulation of GLUT4 protein expression and activation of AMPK. In this study, Metf and Feno were used as positive controls. Furthermore, this study also analyzed the peripheral tissues of STZ-induced mice to determine BB-induced effects on targeted genes. To evaluate the regulatory role of BB on hepatic glucose metabolism, we determined hepatic glucose production genes including phosphoenolpyruvate carboxykinase (PEPCK) and glucose-6-phosphatase (G6 Pase), which are rate-limiting gluconeogenic enzymes [21]. Considering the regulatory role of BB on lipid metabolism, specific changes in hepatic lipogenic fatty acid synthase (FAS) and sterol regulatory element binding protein (SREBP) 1c are to be determined. FAS is a critical focus in fatty acid synthesis [22]. SREBP1c is a key lipogenic transcription factor [23]. To gain insight into hepatic fatty acid oxidation, we quantified PPARα.

## Materials and methods

### Chemicals

Antibodies to GLUT4 (no. sc-79838) were obtained from Santa Cruz Biotech (Santa Cruz, CA, USA), phospho-AMPK (Thr^172^), PPARα (no. ab8934), and PPARγ (no. ab45036) were purchased from Abcam Inc. (Cambridge, MA, USA); FAS (no. 3180). Phospho-Akt (Ser^473^) (no. 4060), total AMPK (Thr^172^), and β-actin (no. 4970) were obtained from Cell Signaling Technology (Danvers MA, USA). Secondary anti-rabbit antibodies were obtained from Jackson ImmunoRes. Lab., Inc. (West Grove, PA, USA).

### Preparation and purification of (−)-Epicatechin-3-O-β-D-allopyranoside

The rhizomes of *Davallia formosana* were obtained from a local market, Yong-Fong Road, Taiping District, Taichung City 41143, Taiwan. These plant rhizomes were identified by the Institute of Chinese Pharmaceutical Sciences, China Medical University, where the voucher specimens (CMPC253) were deposited. The appearance of rhizome is long and creeping, and the connection of petioles and rhizomes is a pinnate slice. The leaves display one to several pinnate cleavages. The extraction and purification of this plant rhizome was performed as previously reported [24]. The pure compound was analyzed by NMR (^1^H, ^13^C; Bruker ADVANCE DPX-200, Germany) and was identified as (−)-epicatechin-3-O-β-D-allopyranoside (BB, the compound of formula (I)) [24], and this BB was provided by Jen Li Biotech Company Ltd., Taiping District, Taichung City, Taiwan. The results of the ^13^C and ^1^HNMR spectrum (200 MHz, CDCl_3_) of BB were in accordance with prior reports [24].

### Cell culture

Myoblast C2C12 cells (ATCC, CRL-1772) were maintained in DMEM supplemented with 10% heat inactivated FBS FBS, 100 U/mL penicillin, and 100 μg/mL streptomycin in a humidified atmosphere with 5% CO_2_ at 37°C. For differentiation into myotubes, cells were reseeded in 9 cm plates at a density of 1x 10^5^cells. After 48 h (over 80% confluence), the medium was switched to DMEM with1% (v/v) FBS and was replaced after 2, 4, and 6 days of culture. The treatments of cells with BB or insulin were initiated on day 6 when myotube differentiation was complete.

### Determination of phospho-Akt (Ser 473)/ total Akt proteins *in vitro*

Supernatant protein concentration was determined via BCA assay (Pierce). Equal amounts of protein were then diluted 4X in SDS sample buffer (62.5 mM Tris-HCl, 20% glycerol, 2% SDS, 75 M DTT, and 0.05% bromophenol blue), subjected to SDS PAGE and were detected by western blotting with antibodies specific for Akt and phospho-Akt Ser^473^. The density blotting was analyzed using Alpha Easy FC software (Alpha Innotech Corp., Randburg, South Africa).

### Animal study

Animal treatments were performed and approved by our Institutional Animal Care and Use Committee (IACUC) (no. CTUST-104-3) and approved by local animal ethics committee (Affidavit of Approval of Central Taiwan Institutional Animal Ethics Committee, permit # P103- I12) S1 Animal Supplement. The study contained two parts of including part 1: Oral glucose tolerance test (OGTT). The ICR mice normal mice (n = 5) were fasted for 12 h but were allowed access to 40, 80, 160 mg/kg BB or an equivalent amount of normal vehicle (water) was given orally 30 min before an oral glucose load (1 g/kg body weight). Blood samples were collected from the retro-orbital sinus of fasting mice at the time of the glucose administration (0) and every 30 minutes until 120 minutes after glucose administration to determine the levels of glucose. The part 2: the 4-week old male C57BL/6J mice (*n* = 55) obtained from the National Laboratory Animal Breeding Center. Streptozotocin (Sigma Chemical, St Louis, MO, USA) was dissolved in 0.05 M cold sodium citrate buffer, pH 4.5. Diabetes was induced by five daily 55 mg/kg intraperitoneal injections of streptozotocin solution over five days, followed by a two-week waiting period. A group of control (CON) mice (*n* = 7) were gavaged identical volumes of vehicle. The STZ mice that were found to develop hyperglycemia, defined as fasting blood glucose above 250 mg/dL, were considered to have diabetes for this study [25]. The STZ-induced diabetic mice (*n* = 48) were then randomly divided into six groups. Three groups were treated with BB at either 10, 20, or 40 mg/kg (groups B1, B2 or B3, respectively). Three comparator groups were treated with similar volumes of either metformin (Metf) (300 mg/kg), fenofibrate (Feno) (250 mg/kg) or distilled water (STZ control group). All treatments were delivered by oral gavage to mice once daily for a period of 28 days. The blood samples (about 150~200 μL) were collected from the retro-orbital sinus of mice after 8 hr fasting (A.M. 8: 00). At the end of the experiment, mice were euthanized using carbon dioxide. Liver, adipose tissue, skeletal muscle, and white adipose tissues (WATs) (including mesenteric and retroperitoneal WAT) were excised and weighed and immediately stored at −80°C until use. Aside from blood glucose, heparin (30 units/ml) (Sigma) were firstly added into the collecting blood tubes, and followed by drying of tubes, and we immediately used the heparin–processed tubes to collect blood samples. Plasma samples were collected via centrifugation of whole blood at 1600 **g** for 15 minutes at 4°C, followed by separation within 30 minutes. The supernatant were obtained for total cholesterol (TC) and triglyceride (TG) analysis (individually 20~30 μL). Aliquots of plasma samples (>25 μL) were obtained for insulin, leptin, and adiponectin. Body weight was daily measured (A.M. 10: 00) at the same time throughout the study. And at the meanwhile, animals monitoring was done, including body weight changing, skin disease, food consumption, and the appearance of the animals. Body weight gain is considered as the difference between one day and the next day. The amount of pellet food was weighed, and followed by weighing the amount of remaining food after 24 h. The difference is represented the daily food intake.

### Measurement of blood glucose, biochemical parameters, and adipocytokine levels

Blood samples were collected from the retro-orbital sinuses of 8 hr fasted mice, and the samples (> 20 μL) immediately on tinfoil paper and quickly on machine for analysis of blood glucose levels. Blood glucose levels were measured by the glucose oxidase method (Model 1500; Sidekick Glucose Analyzer; YSI Incorporated, Yellow Springs, USA). The levels of TG and TC were measured using commercial assay kits in accordance with manufacturer directions (Triglycerides-E test and Cholesterol-E test, Wako Pure Chemical, Osaka, Japan). Aliquots of plasma samples (>25 μL) were used for insulin, leptin, and adiponectin levels by enzyme-linked immunosorbent assay (ELISA) kits (mouse insulin ELISA kit, Mercodia, Uppsala, Sweden; mouse leptin ELISA kit, Morinaga, Yokohama, Japan) as previous procedures [24–26]. Percent HbA1c was measured with a Hemoglobin A_1C_ kit (BioSystems S.A., Barcelona, Spain).

### Histopathology examinations

Parts of liver and pancreas specimens were fixed with formalin (200 g/kg) neutral buffered solution and embedded in paraffin and sections (8 μm) were cut and stained with hematoxylin and eosin and images were photographed in accordance with previous reports [24–26]. Ballooning degeneration is investigated in the liver of mice. This phenomena is associated with hepatocyte death and glycogen accumulation in the center, and the nucleolus was squeezed into the other side, implying that the ballooning degeneration.

### Relative quantization of mRNA and Western blotting

Relative mRNA quantification (primers are described in Table 1) and immunoblots for measurement of muscle membrane GLUT4 and hepatic phospho-AMPK (Thr^172^) and phospho-Akt (Ser^473^) proteins were performed as described in previously reported studies [24–26]. Liver tissue was analyzed for PPARα and FAS protein content. Adipose tissue was analyzed for PPARγ and FAS protein content. Skeletal muscle from mice is subjected to analyses of GLUT4 protein expressions, and total membrane fraction was collected and determined by a described procedure [27,28**]. Skeletal muscle** was powdered under liquid nitrogen and homogenized in buffer (pH 7.4) containing 250 mmol/L sucrose, 50 mmol/L Tris, and 0.2 mmol/L edetic acid for 20 s. The homogenate was centrifuged at 9,000 × *g* for 10 min (4°C) and the supernatant was reserved. The pellets were cleaned with buffer and centrifuged for three times. All three supernatants were mixed and centrifuged at 190,000 × *g* for 60 min (4°C). The resulting pellet was resuspended in a small amount of buffer (about 0.5 mL) as a total **membrane** fraction [27,28]. The expression levels of GLUT4, phospho-AMPK, and total AMPK were determined by Western blotting as previously described [25,26].

**Table 1:** Primers used in this study.

Gene	Accession number	Forward primer and reverse primer	PCR product (bp)	Annealing temperature (°C)
Liver				
PEPCK	NM_011044.2	F: CTACAACTTCGGCAAATACC	330	52
		R: TCCAGATACCTGTCGATCTC		
G6Pase	NM_008061.3	F: GAACAACTAAAGCCTCTGAAAC	350	50
		R: TTGCTCGATACATAAAACACTC		
11β-HSD1	NM_008288.2	F:AAGCAGAGCAATGGCAGCAT	300	50
		R: GAGCAATCATAGGCTGGGTCA		
PPARα	NM_011144	F: ACCTCTGTTCATGTCAGACC	352	55
		R: ATAACCACAGACCAACCAAG		
SREBP1c	NM_011480	F: GGCTGTTGTCTACCATAAGC	219	50
		R: AGGAAGAAACGTGTCAAGAA		
CPT1a	BC054791.1	F:CTTGTGACCCTACTACATCC	332	51
		R:TCATAGCAGAACCTTAATCC		
aP2	NM_024406	F:TCACCTGGAAGACAGCTCCT	142	52
		R:TGCCTGCCACTTTCCTTGT		
SREBP2	AF289715.2	F: ATATCATTGAAAAGCGCTAC	256	48
		R: ATTTTCAAGTCCACATCACT		
GAPDH	NM_031144	F: TGTGTCCGTCGTGGATCTGA	99	55
		R: CCTGCTTCACCACCTTCTTGA		

### Statistics

Results are presented as the means +/- standard errors. Comparisons among groups were performed using ANOVA and were coupled with Dunnett’s tests. All P-values less than 0.05 were considered to be significant.

## Results

### Phospho-Akt (Ser 473)/ total Akt proteins *in vitro*

**Fig 2:**
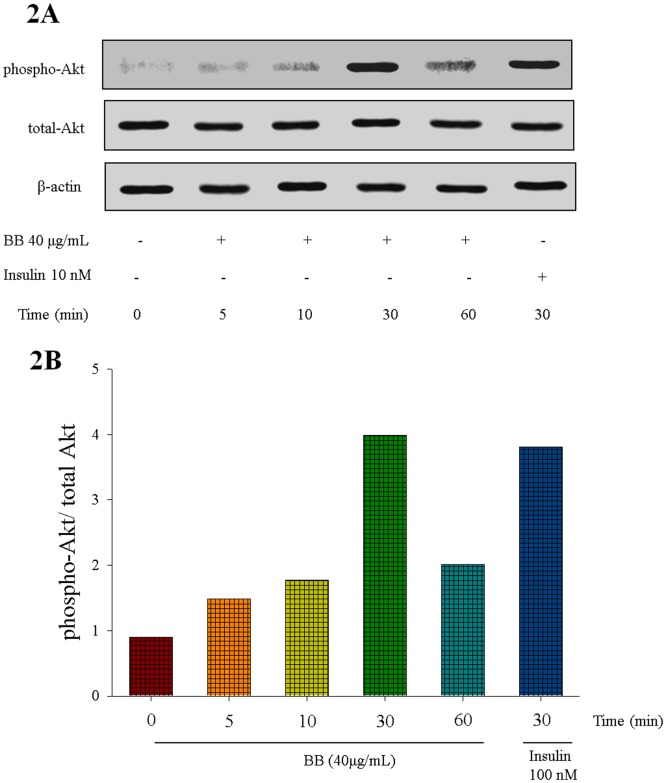
(−)-epicatechin-3-O-β-D-allopyranoside (BB) activates Akt signaling pathways. The cell lysates were analyzed via Western blotting for p-Akt and t-Akt. (A) representative image; Akt phosphorylation was determined from C2C12 cells, and treated with 40 μg/ mL of BB for the indicated period of time (5–60 min); (B). The ratios of phospho-Akt to total Akt forms were analyzed and presented phosphorylation of Akt.

At the end, BB was shown to activate Akt in a time-dependent manner and this was comparable to that of insulin. The phosphorylation of Akt reached a maximum level at 30 min and then decreased to basal level at 60 min of BB treatment (Fig 2A and 2B). S1–S3 Figs.

### Oral glucose tolerance test

**Fig 3:**
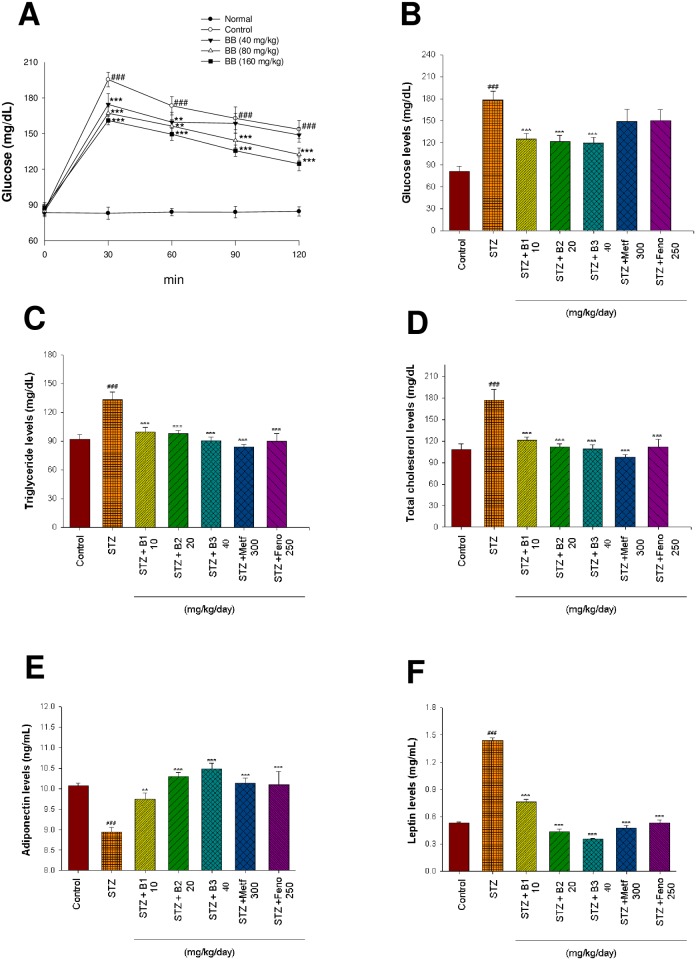
Effects of (−)-epicatechin-3-O-β-D-allopyranoside (BB) including two parts. (A) oral glucose tolerance (OGTT). OGTT test was performed on 12 h fasted ICR mice (n = 5) that were allowed access to 40, 80, and 160 mg/kg BB or an equivalent amount of vehicle (water), which were given orally 30 min before an oral glucose load (1 g/kg body wt). The control group was given glucose, whereas the normal group was not. Blood samples were collected from the retro-orbital sinus of fasted mice at the time of the glucose administration (0) and every 30 min until 120 minutes after glucose administration and the blood glucose level was monitored. (B)~(F): Effects of BB on streptozotocin (STZ)-induced diabetic mice; (B) blood glucose levels, (C) triglycerides levels, (D) total cholesterol levels, (E) adiponectin levels, and (F) leptin levels at week 4 by oral gavage (−)-epicatechin-3-O-β-D-allopyranoside (BB: B1, B2, and B3, 10, 20 and 40 mg/kg body weight), or metformin (Metf; 300 mg/kg body weight), or fenofibrate (Feno; 250 mg/kg body weight) in streptozotocin (STZ)-induced mice. All values are means ± SE (n = 9). ^#^ P < 0.05, ^##^ P < 0.01, and ^###^ P < 0.001 compared with the control (CON) group; * P < 0.05, ** P < 0.01, and *** P < 0.001 compared with the streptozotocin plus vehicle (distilled water) (STZ) group by ANOVA.

As shown in Fig 3A, following treatment with 40 mg/kg BB, the levels of blood glucose were significantly decreased at 30 and 60 min, and treatment with 80 and 160 mg/kg BB, the levels of blood glucose were significantly decreased at 30, 60, 90 and 120 min glucose-loading as compared with the control.

### Body weight, body weight gain, food intake, and tissue weight

During the experiment, because of STZ-induction leads to 5 mice death including the STZ group (*n* = 2), B2 group (*n* = 1), Metf group (*n* = 1), and Feno group (*n* = 1), and 5 mice died unexpectedly when they were found dead. As shown in Table 2, the final body weights of vehicle-treated STZ mice had decreased body weigh as compared with the CON group (P < 0.05). B3- and Metf-treated groups had decreased body weights as compared with the STZ group (P < 0.001, P < 0.01, respectively). The body weight gain of STZ group did not differ from the CON group. B2-, B3-, Metf-, and Feno-treated group decreased body weight gain as compared with the STZ group. Food intake of the STZ group was increased as compared with the CON group (P < 0.05). Additionally, mice in groups B1, B2, B3, Metf, and Feno had decreased food intake as compared to the vehicle-treated STZ mice. The absolute epididymal white adipose tissue (EWAT), skeletal muscle, BAT, and liver weights of STZ mice did not differ from the CON mice. The STZ mice also showed a decrease in retroperitoneal WAT (RWAT) weight compared to the CON group (P < 0.01). The B3-treated mice increased the weight of EWAT, while Metf-treated mice decreased the weights of EWAT and increased the weights of retroperitoneal WAT (RWAT) as compared with the STZ group. Feno treatment resulted in increased liver weight in comparison to the STZ group (P < 0.001) (Table 2).

**Table 2:** Effects of (−)-Epicatechin-3-O-β-D-allopyranoside (BB) on absolute tissue weight, body weight, body weight gain, and food intake^a^ in streptozotocin-induced diabetic mice.

Parameter	CON	STZ	STZ+B1	STZ+B2	STZ+B3	STZ+Metf	STZ+Feno
			10^a^	20^a^	40^a^	300^a^	250^a^
Absolute tissue weight (g)							
EWAT	0.293 ± 0.010	0.421 ± 0.190	0.563 ± 0.007*	0.507 ± 0.032	0.388 ± 0.037	0.290 ± 0.041*	0.516 ± 0.069
RWAT	0.047 ± 0.008	0.026 ±0.003^##^	0.013 ± 0.002	0.020 ± 0.003	0.023 ± 0.002	0.042 ± 0.005*	0.015 ± 0.003
Skeletal muscle	0.254 ± 0.022	0.220 ± 0.017	0.276 ± 0.013	0.240 ± 0.01	0.268 ± 0.034	0.260 ± 0.047	0.244 ± 0.031
BAT	0.086 ± 0.009	0.089 ± 0.005	0.086 ± 0.006	0.086 ± 0.002	0.081 ± 0.005	0.095 ± 0.005	0.078 ± 0.007
Liver	0.841 ± 0.015	0.862 ± 0.017	0.869 ± 0.022	0.867 ± 0.045	0.827 ± 0.016	0.809 ± 0.015	1.119 ± 0.038*
weight gain (g)	1.23 ± 0.23	1.34 ± 0.20	1.58 ± 0.48	0.41 ± 0.19*	0.12 ± 0.38**	0.08 ± 0.31**	-0.35 ± 0.76*
Final body weight (g)	22.55 ± 0.29	21.66 ± 0.27^#^	22.10 ± 0.50	21.77 ± 1.00	19.93±0.31***	20.32 ± 0.26**	20.33 ± 0.97
food intake (g/day/mouse)	3.64 ± 0.06	3.87 ± 0.04^#^	3.40 ± 0.03***	3.56 ± 0.04***	3.39 ± 0.04***	3.42 ± 0.04***	3.40 ± 0.09***
HbA_1C_ (%)	6.1 ± 0.5	10.8 ± 3.5^#^	7.5 ± 0.8*	7.1 ± 0.7**	6.8 ± 0.6***	8.2 ± 4.6	8.5 ± 3.9
Insulin (μg/L)	1.071 ± 0.014	0.754±0.009^###^	0.835 ± 0.031	0.906 ± 0.009*	0.945 ±0.011*	0.857 ± 0.043	0.860 ± 0.045

All values are means ± SE (n = 9).

^#^ P < 0.05,

^##^ P < 0.01, and

^###^ P < 0.001 compared with the control (CON) group;

* P < 0.05,

** P < 0.01, and

*** P < 0.001 compared with the streptozotocin (STZ) plus vehicle (distilled water) (STZ) group.

(−)-epicatechin-3-O-β-D-allopyranoside (BB): B1: 10, B2: 20, B3: 40 mg/kg body weight; Metf: metformin (300 mg/kg body weight); Feno: fenofibrate (250 mg/kg body weight). BAT, brown adipose tissue; RWAT, retroperioneal white adipose tissue.

^a^Dose (mg/kg/day)

### Blood glucose, HbA_1C_, insulin, triglyceride, total cholesterol, leptin, and adiponectin levels

STZ-induced mice had higher blood glucose and HbA_1C_ levels than did the CON mice, whereas plasma insulin levels were lower in the STZ-treated group compared with the CON group (P < 0.001, P < 0.01, and P < 0.001; respectively). The B1-, B2-, and B3-treated mice had significantly decreased HbA_1C_ levels (Table 2). The B1-, B2-, and B3- treated mice had significantly lower blood glucose levels (Fig 3B). B2 and B3-treated groups increased plasma insulin levels as compared to the STZ group (Table 2). Plasma triglyceride (TG) and total cholesterol (TC) concentrations were higher in the STZ group compared to the CON group (P < 0.001 and P < 0.001, respectively). Plasma TG and TC concentrations were reduced by B1, B2, B3, Metf, and Feno treatment (Fig 3C and 3D). STZ induction reduced plasma adiponectin concentrations as compared to the CON group (P < 0.001). Treatment with B1, B2, B3, Metf, or Feno significantly raised adiponectin levels as compared to the STZ group (P < 0.01, P < 0.001, P < 0.001, P < 0.001 and P < 0.001, respectively) (Fig 3E). Plasma leptin levels were higher in the STZ group than in the CON group (P < 0.001). B1-, B2-, B3-, Metf- and Feno- treated groups had significantly lower leptin levels compared to the STZ group (P < 0.001, P < 0.001, P < 0.001, P < 0.001 and P < 0.001, respectively) (Fig 3F).

### Histology

**Fig 4:**
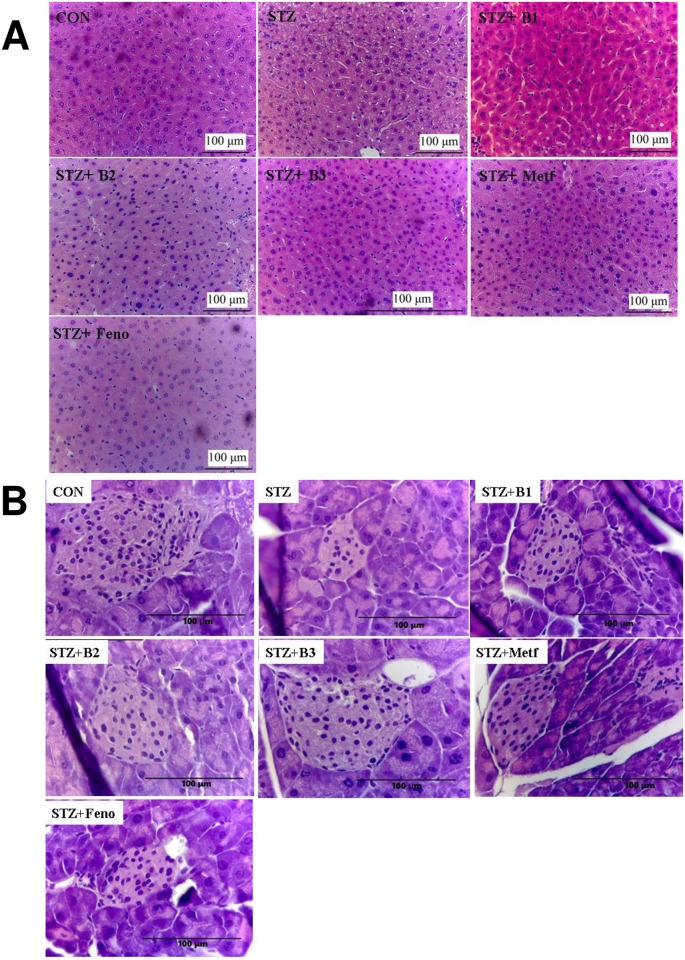
Histology examinations. (A) liver tissue and (B) pancreatic islets of Langerhans of mice in the control (CON), streptozotocin plus vehicle (distilled water) (STZ), STZ + B1, STZ + B2, STZ + B3, STZ + metformin (Metf), or STZ + fenofibrate (Feno) groups by hematoxylin and eosin-staining. Magnification: 10 (ocular) × 20 (object lens). (−)-epicatechin-3-O-β-D-allopyranoside (BB): B1: 10, B2: 20, B3: 40 mg/kg body wt; Metf: metformin (300 mg/kg body wt); Feno: fenofibrate (250 mg/kg body wt).

The STZ-treated group showed a slight ballooning of hepatocytes compared with the CON group. However, hepatocytes displayed no ballooning after treatment with B1, B2, B3, Metf, or Feno. These results strongly suggest that BB did not cause hepatic TG accumulation (Fig 4A). Langerhans islet was found in the field of vision with a light color. The control mice showed that the islets were round and without cell distortion. The vehicle-treated STZ mice showed with smaller size and number of Langerhans islet cells. Treatment with B2 and B3 showed improvement in size and number of Langerhans islet cells (Fig 4B).

### mRNA levels of targeted hepatic glucose and lipid metabolism regulatory genes

**Fig 5:**
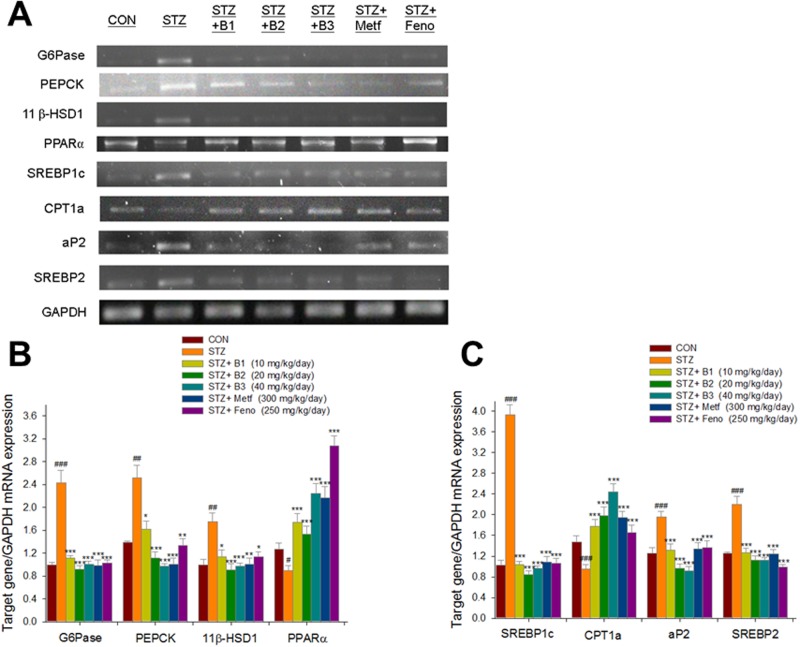
Semiquantative RT-PCR analysis on targeted gene mRNA levels in liver tissue of the mice by oral gavage (−)-epicatechin-3-O-β-D-allopyranoside (BB) (B1, B2, and B3, 10, 20 and 40 mg/kg body weight), or metformin (Metf; 300 mg/kg body weight), or fenofibrate (Feno; 250 mg/kg body weight). (A) representative image; (B, C) quantification of the ratio of target gene to GAPDH mRNA expression. Total RNA (1 μg) isolated from tissue was reverse transcripted by MMLV-RT; 10 μL of RT products were used as templates for PCR. The expression levels of G6Pase, PEPCK, 11β-HSD1, PPARα, SREBP1c, CPT1a, aP2, and SREBP2 mRNA were measured and quantified by image analysis. Values were normalized to GAPDH mRNA expression. All values are means ± SE (n = 9). ^#^ P < 0.05, ^##^ P < 0.01, and ^###^ P < 0.001 compared with the control (CON) group; * P < 0.05, ** P < 0.01, and *** P < 0.001 compared with the streptozotocin plus vehicle (distilled water) (STZ) group.

The STZ-induced mice expressed higher mRNA levels of glucose-6-phosphatase (G6Pase), phosphoenolpyruvate carboxykinase (PEPCK), 11beta hydroxysteroid dehydrogenase (11β-HSD1), sterol regulatory element binding protein 1c (SREBP1c), adipocyte fatty acid binding protein 2 (aP2) and sterol regulatory element binding protein 2 (SREBP2). In the same mice, levels of PPARα and carnitine palmitoyl transferase Ia (CPT1a) were reduced. The B1-, B2-, B3-, Metf- and Feno- treated mice expressed lower mRNA levels of G6Pase, PEPCK, 11β-HSD1, SREBP1c, aP2 and SREBP2. The B1-, B2-, B3-, Metf- and Feno- treated mice expressed higher mRNA levels of PPARα and CPT1a (Fig 5A and 5B).

### Expressions of GLUT4, phosphorylation of Akt and AMPK, PPAR alpha and gamma, and FAS in the liver and skeletal muscle tissues

**Fig 6:**
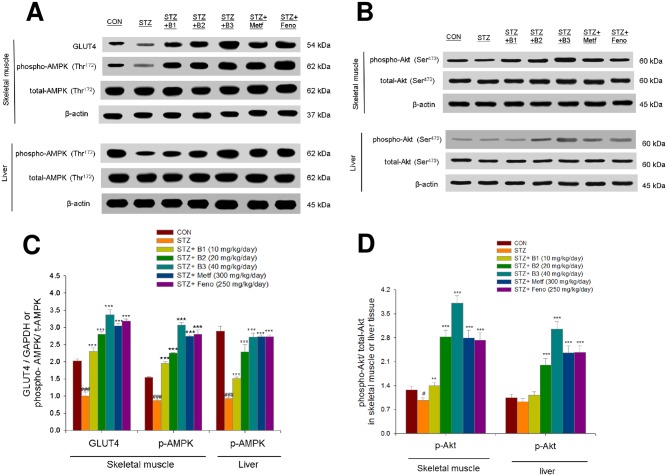
The protein contents of (A) GLUT4 in skeletal muscle, and phospho-Akt (Ser^473^)/total Akt, phospho-AMPK (Thr^172^) /total AMPK in both skeletal muscle and liver tissue of the mice by oral gavage (−)-epicatechin-3-O-β-D-allopyranoside (BB). (A, B) representative image; (C, D) quantification of the GLUT4 expression levels and the ratio of phospho-Akt to total Akt and phospho-AMPK to total AMPK (mean ± SE, *n* = 9). Protein was separated by 12% SDS-PAGE detected by Western blot. ^#^ P < 0.05, ^##^ P < 0.01, and ^###^ P < 0.001 compared with the control (CON) group; * P < 0.05, ** P < 0.01, and *** P < 0.001 compared with the streptozotocin plus vehicle (distilled water) (STZ) group. B1, B2, and B3, (−)-epicatechin-3-O-β-D-allopyranoside (BB) (B1, B2, and B3, 10, 20, and 40 mg/kg body weight, respectively); metformin (Metf; 300 mg/kg body weight); fenofibrate (Feno, 250 mg/kg body weight).

**Fig 7:**
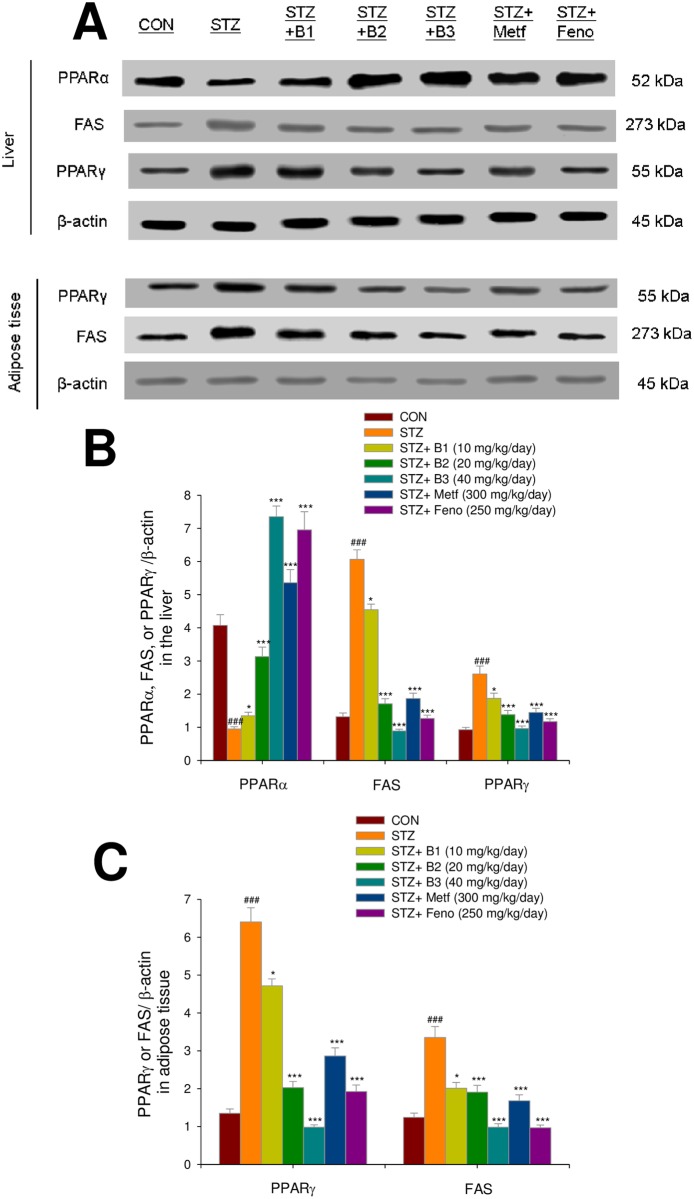
Expression levels of PPARα, FAS, and PPARγ in the liver and PPARγ and FAS in adipose tissue of mice by oral gavage (−)-epicatechin-3-O-β-D-allopyranoside (BB). (A) representative image; (B, C) quantification of the expression levels of PPARα, FAS, and PPARγ in the liver and expression levels of FAS and PPARγ in adipose tissue. Protein was separated by 12% SDS-PAGE detected by Western blot. All values are means ± SE (*n* = 9). (−)-epicatechin-3-O-β-D-allopyranoside (BB): B1: 10, B2: 20, B3: 40 mg/kg body wt; Metf: metformin (300 mg/kg body wt); Feno: fenofibrate (250 mg/kg body weight). (−)-epicatechin-3-O-β-D-allopyranoside (BB): B1: 10, B2: 20, B3: 40 mg/kg body weight; Metf: metformin (300 mg/kg body wt); Feno: fenofibrate (250 mg/kg body weight).

The STZ-induced mice expressed lower levels of striated muscle membrane GLUT4 as compared with the CON group (P < 0.01). Striated muscle membrane GLUT4 levels were higher in the B1-, B2-, B3-, Metf- and Feno-treated groups as compared with the STZ group. The STZ-induced mice expressed lower levels of phospho-AMPK/total AMPK in both muscle and liver tissue. In both skeletal muscle and liver tissue, the B1-, B2-, B3-, Metf- and Feno- treated groups expressed higher levels of phospho-AMPK/total AMPK compared with the STZ- treated group. The STZ-induced mice expressed lower levels of phospho-Akt/total Akt in skeletal muscle, and the B1-, B2-, B3-, Metf- and Feno- treated groups expressed higher levels of phospho-Akt/total Akt, and the B2-, B3-, Metf- and Feno-treated groups expressed higher levels of phospho-Akt/total Ak in liver tissue compared with the STZ- treated group (Fig 6A, 6B, 6C and 6D). S4–S16 Figs. After STZ-induction, the STZ group expressed lower levels of hepatic PPARα and higher levels of FAS and PPARγ than did the CON group. Following treatment, the B1-, B2-, B3-, Metf-, and Feno-treated mice expressed higher levels of PPARα and lower levels of FAS and PPARγ. STZ-induction increased expressed levels of PPARγ and FAS. Finally, B1-, B2-, B3-, Metf- and Feno- treatment reduced the expressed levels of PPARγ and FAS (Fig 7A, 7B and 7C). S17–S23 Figs.

## Discussion

The aim of this study was to evaluate whether BB could reduce blood glucose levels and improve the lipid profile of STZ-induced diabetic mice. In this study, STZ-injections resulted in hyperglycemia, similar to the results of a previously reported study [29]. BB treatment resulted in a significant decrease in blood glucose levels. Chronic hyperglycemia in diabetes mellitus can result in excess free radical production and oxidative stress, thus led to the development of diabetic neuropathy and a variety of diabetic complications are known to act in regulation with high oxidative stress levels [30,31]. The STZ-induction display the increased levels of glycosylated hemoglobin, which is a marker with relation of surrounding glycemia during 2–3 month, implied the oxidation and damage in tissues [32,33]. In this study, STZ-induced diabetic mice increased HbA1_C_ levels showing that oxidative damage. BB treatment caused a reduction of increased HbA_1C_ levels, implying that BB may prevent oxidative damage caused by the glycation reaction in diabetic states. In this study, STZ-induction caused a decrease in body weights, and this was in line with previous study [34]. Our STZ-induced diabetic mice exert a decrease in insulin levels but an increase in food intake, and these were in agreement with other study [35], suggesting that insulin deficiency in STZ-induced diabetic rodents results in the subsequent increase in food intake with relation to hypothalamic AMPK activation and this remains further to be studied. There is no significant difference on organ weights, and these discrepancies possibly reflect variation in experimental models, species differences, doses of STZ, and duration of STZ exposure, and require further study. BB caused a significant increase in insulin levels (B2 and B3 groups) and decreased in blood glucose and HbA1_C_ levels. Our study demonstrated that BB exerts antidiabetic activity in STZ-induced diabetic mice and OGTT test. The ability of BB to reduce blood glucose and HbA_1C_ levels in diabetic mice is due to its potential of secreting insulin from the existing islet β-cells. Moreover, many polyphenols are shown to exhibit an antioxidant activity, and our results showed that the antioxidant property of BB displayed the enhancement of Akt phosphorylation not only *in vivo* but also in C2C12 myotubes and activated Akt pathway, similar to insulin (Fig 2).

This plant “Gusuibu” is reported to contain polyphenol contents and display antioxidant activities [10]. Moreover, polyphenol is defined by the aromatic ring with more than two hydroxyl groups, and thus BB belongs to one of polyphenols. Polyphenols could protect cell against oxidative stress by decreases in reactive oxygen species (ROS) or by enhancement in antioxidant defense systems [6]. STZ-induction caused a production of ROS and decreased antioxidant enzyme activity including superoxide dismutase (SOD) and catalase, especially in the pancreatic tissue [7]. Our results are in agreement with the previous study showing that the diabetic islets showed retraction from their classic round-shaped compared to the control islets [36], and these suggest that circulating ROS generated by STZ on β-cell. Antioxidants have been suggested to afford protection to the pancreas against oxidative stress in diabetes mellitus [37]. Our results here showed that BB exhibited improvement in islets size and less degeneration, and suggesting that BB may act by the antioxidant activities (within the pancreas) with an increase in radical scavenging and a decrease in ROS in STZ-treated β-cell, and this protection of the pancreas against oxidative stress and will consequently contribute to its hypoglycemic effects.

A single large dose (180–190 mg/kg body weight of STZ causes massive β cell necrosis within 2–3 days, whereas a multiple low dose (MLD)-STZ injections (35–55 mg/kg mg/kg bodyweight for 4–5 consecutive days) are often used to model destruction of pancreatic β cells, with a 70% reduction of the islet per pancreas area [38]. In this study, fasting blood glucose was significantly increased in vehicle-treated STZ mice as compared to the CON mice, implying that STZ treatment caused β cell mass reduction, and followed by insulin insufficiency, in turn, thus resulting in hyperglycemia. BB significantly reduced glucose levels, with increases (B2 and B3 groups) in plasma insulin levels. In this study, Metf and Feno displayed no effects in glycemic control, with less increase (not statistically significant) in plasma insulin levels. The results presented here suggest that there are different mechanisms among these treatments. Although metformin is claimed to upregulate the synthesis of insulinotropic agents such as glucagon like peptide-1 (GLP-1) through a mechanism requiring PPARα [39] and it was suggested to be well suited for combination with incretin-based therapies [39], there is a majority of reduction in beta cell mass and insulin insufficiency in MLD-STZ induced type 1 diabetic mice, and thus metformin has no effects in glycemic control. Metformin may have minor glucose-induced insulinotropic effects via stimulation of GLP-1 but is not an insulin secretagogue just like fenofibrate. These results presented here suggest that BB exerts blood glucose-lowering effects via insulin secretagogue *in vitro* and *in vivo* or anti-oxidant effects not only by HbA1_C_ levels but also within the pancreas.

Our present results show that STZ-induced diabetic mice displayed elevated levels of circulating triglycerides and total cholesterol, as was reported in earlier studies [40]. These effects were reversed by BB treatment. BB exhibited a triglyceride-lowering effect (24.7%–32.0%) and total cholesterol-lowering effect (31.6%–38.4%). These results imply that BB, Metf and Feno reduced blood triglyceride and total cholesterol levels by inhibition of FAS and enhancement of PPARα expression levels in the liver. The results also imply that BB may have therapeutic potential in the management of type 1 diabetes accompanied with hypertriglyceridemia.

The promoted glucose uptake into skeletal muscle is proposed to include two pathways. One is insulin-dependent mechanisms lead to activation of Akt [16]. The other is contraction- or AICAR- mediated stimulation of AMPK [16]. In skeletal muscle, both of the two pathways also phosphorylate the protein AS160 to increase translocation of GLUT4 to the plasma membrane [41]. Akt (PKB) stimulates glucose uptake by modulating glucose transporter 4 (GLUT4) [42]. This study was to evaluate the antidiabetic molecular mechanisms of BB by measurement of membrane GLUT4 expression levels in skeletal muscle because skeletal muscular GLUT4 accounts for the majority of glucose uptake. Membrane GLUT4 protein levels were shown to be suppressed in STZ-induced mice, as was demonstrated in a previous study [43]. Administration of BB caused a 1.29–2.35-fold increase in muscular membrane GLUT4 expression as compared to the vehicle-treated STZ group. In this study, BB treatment in C2C12 myotubes at 30 min increased insulin levels and enhanced the phosphorylation of Akt. Moreover, in STZ-induced diabetic mice, administration of BB increased the expression of phospho-Akt/ total Akt and phospho-AMPK/total AMPK in skeletal muscle. These results indicate that BB may play a role in myotube and skeletal muscle through insulin-Akt or /and AMPK pathways. BB could stimulate glucose transport activity partly by insulin pathway and partly by AMPK activation and providing a therapeutic approach to treat diabetes.

Both insulin and phosphorylation of AMPK inhibit mRNA and protein of phosphoenolpyruvate carboxykinase (PEPCK) and glucose-6-phosphatase (G6Pase) gene transcription [44,45]. Insulin and glucagon regulate the expression of a variety of proteins (including the key target PEPCK) to maintain blood glucose with normal limits. PEPCK expression is induced by glucagon, but is inhibited by glucose-induced increases in insulin secretion [45]. The regulation of gluconeogenesis by insulin is complex, and insulin can inhibit this pathway by suppressing the transcription of the enzyme PEPCK [46]. The effects of glucose and insulin on PEPCK expression are additive, by inhibiting PEPCK gene transcription, glucose participate in a feedback control loop that governs its production from gluconeogenesis [47]. Insulin inhibits gluconeogenesis via Akt-dependent phosphorylation of FoxO1, in turn, to inhibition of PEPCK and G 6Pase gene transcription [48]. In this study, BB increases plasma insulin levels and enhances phosphorylation of Akt, suggesting that a molecular mechanism of BB might be partly via insulin- Akt-FoxO1 to inhibition of PEPCK pathway remains further to be clarified.

This study was to examine the antihyperlipidemic effects and mechanisms of BB. PPARα agonists have been proposed as a breakthrough treatment to reduce blood triglyceride levels [21]. Fenofibrate is one of the PPARα agonists that may have this effect [49]. CPT-1a prepares fatty acids for mitochondrial oxidation. It has been considered to be the rate-limiting enzyme in this process [50]. Administration of BB increased expressed levels of PPARα (Fig 7A and 7B) and CPT1a mRNA (Fig 5A and 5C), implying that enhancement of β-oxidation prevented the accumulation of hepatic triacylglycerol. Furthermore, PPARα-deficient mice displayed dysregulated SREBP-mediated lipogenic genes [51]. SREBP-1c is a key lipogenic transcription factor that stimulates lipogenic enzyme expression and contributes to fatty acid synthesis and TG accumulation [52]. Furthermore, adipocyte fatty acid binding protein 2 (aP2) deficiency in murine models has been shown to protect against the development of dyslipidemia, hyperglycemia, insulin resistance and fatty liver disease in both genetic and dietary obesity [53]. Ablation of aP2 and mall (a fatty acid binding protein similar to aP2) increases hepatic accumulation of longer-chain fatty acids, thus resulting in decreased expression of SREBP1c and several downstream lipogenic enzymes [53]. In the present study, BB-treated mice expressed reduced hepatic mRNA levels of aP2 and lipogenic SREBP1c, which also led to reduced hepatic triglyceride output. In turn, this reduced blood TG. Furthermore, because SREBP2 plays a core role in modulating total cholesterol synthesis [54], it appears that the demonstrated reduction of circulating total cholesterol by BB is caused by inhibition of total cholesterol synthesis.

Administration of BB, Feno and Metf reduced adipose tissue expression of FAS protein and PPARγ, which stimulates adipogenesis and lipogenesis in adipocytes [55]. Furthermore, adipogenesis and fatty acid synthesis and lipid accumulation was found to be decreased in adipose tissue. These results suggest that BB may remove fat from adipose tissue to peripheral tissues through the mechanisms of increased hepatic lipid catabolism and decreased adipocyte adipogenesis and FAS in adipose tissue. This leads to reduced TG levels in the liver, blood, and adipose tissue, as demonstrated by the near-absence of hepatic lipid droplets.

Circulating lipids are fluctuating among blood, the liver tissue, and adipose tissue. Treatment with globular domains of adiponectin has been shown to enhance glucose uptake and fatty acid oxidation, and is negatively associated to plasma lipid makers [56]. BB treatment increased the levels of adiponectin but decreased leptin. PPARγ and leptin are major factors that play a key role in influencing adipocyte differentiation and adipose tissue metabolism [57]. According to previous reports [56,57], the effects of BB on glucose and lipid metabolism might be associated with adiponectin and /or leptin secretion.

## Conclusion

**Fig 8:**
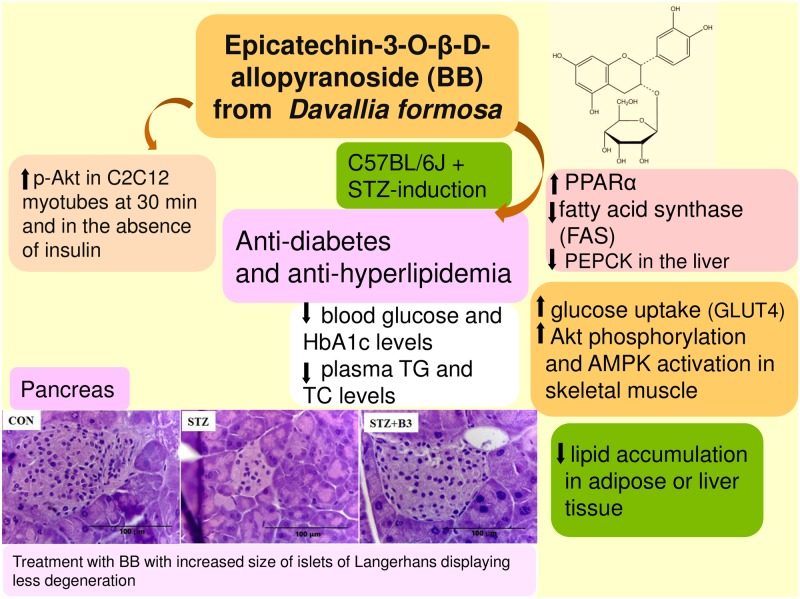
The graphic abstract of (−)-epicatechin-3-O-β-D-allopyranoside (BB) in streptozotocin-induced diabetic mice.

BB belongs to one of polyphenols which had been proposed to exhibit antioxidant activity. The STZ-induced type 1 diabetes mellitus mice of the present study showed that well-known diabetes associated with reactive oxidant species within the pancreas. In summary (Fig 8), BB treatment may have the ability to lower blood glucose and triglyceride levels, thereby ameliorating hyperglycemia and hypertriglyceridemia. The STZ-induced diabetic islets showed retraction from their classic round-shaped as compared with the control islets. The BB-treated groups showed improvement in islets size and less degeneration. In addition to BB’s role in enhancement of phosphorylation of Akt in the absence of insulin in C2C12 myotube, suggesting that BB act as a regulation of insulin pathway (Akt), BB significantly increased the expression of phosphorylation of Akt and AMPK and membrane GLUT4 in the skeletal muscle of STZ-treated mice; moreover, with inhibition of PEPCK and G6Pase mRNA levels in liver tissue, and combination of these effects contribute to BB’s antidiabetic activity. These results demonstrated that BB exhibited the antioxidant activity by the decreases in HbA1_C_ levels and histological examination within pancreas. BB treatment enhanced hepatic phosphorylation of AMPK and Akt in liver tissue. BB enhanced the expression of fatty acid oxidation genes including PPARα, and reduced the expression of lipogenic FAS and hepatic SREBP1c mRNA, with the net effect of reducing blood triglyceride levels. Our results indicate that BB exhibits favorable potential for the treatment of type 1 diabetes coincident with hyperlipidemia.

## Supporting information

S1 Animal Supplement Animal ethics. (PDF)

S1 Fig Western blotting for phospho-Akt in C2C12 myotubes in Fig 2A. Representative image; Akt phosphorylation was determined from C2C12 cells, and treated with 40 μg/ mL of BB for the indicated period of time (5–60 min).
(TIF)

S2 Fig Western blotting for total-Akt in C2C12 myotubes in Fig 2A. Representative image; total-Akt was determined from C2C12 cells, and treated with 40 μg/ mL of BB for the indicated period of time (5–60 min).
(TIF)

S3 Fig Western blotting for β-actin in C2C12 myotubes in Fig 2A. Representative image; β-actin was determined from C2C12 cells, and treated with 40 μg/ mL of BB for the indicated period of time (5–60 min).
(TIF)

S4 Fig Western blotting for muscular membrane GLUT4 in Fig 6A. Representative image; the skeletal muscle was obtained from the STZ-induced diabetic mice following treatment with vehicle, BB, Metf, or Feno for 4 weeks.
(TIF)

S5 Fig Western blotting for muscular phospho-AMPK in Fig 6A. Representative image; the skeletal muscle was obtained from the STZ-induced diabetic mice following treatment with vehicle, BB, Metf, or Feno for 4 weeks.
(TIF)

S6 Fig Western blotting for muscular total-AMPK in Fig 6A. Representative image; the skeletal muscle was obtained from the STZ-induced diabetic mice following treatment with vehicle, BB, Metf, or Feno for 4 weeks.
(TIF)

S7 Fig Western blotting for muscular β-actin in Fig 6A. Representative image; the skeletal muscle was obtained from the STZ-induced diabetic mice following treatment with vehicle, BB, Metf, or Feno for 4 weeks.
(TIF)

S8 Fig Western blotting for hepatic phospho-AMPK in Fig 6A. Representative image; the liver tissue was obtained from the STZ-induced diabetic mice following treatment with vehicle, BB, Metf, or Feno for 4 weeks.
(TIF)

S9 Fig Western blotting for hepatic total-AMPK in Fig 6A. Representative image; the liver tissue was obtained from the STZ-induced diabetic mice following treatment with vehicle, BB, Metf, or Feno for 4 weeks.
(TIF)

S10 Fig Western blotting for hepatic β-actin in Fig 6A. Representative image; the liver tissue was obtained from the STZ-induced diabetic mice following treatment with vehicle, BB, Metf, or Feno for 4 weeks.
(TIF)

S11 Fig Western blotting for muscular phospho-Akt in Fig 6B. Representative image; the skeletal muscle was obtained from the STZ-induced diabetic mice following treatment with vehicle, BB, Metf, or Feno for 4 weeks.
(TIF)

S12 Fig Western blotting for muscular total-Akt in Fig 6B. Representative image; the skeletal muscle was obtained from the STZ-induced diabetic mice following treatment with vehicle, BB, Metf, or Feno for 4 weeks.
(TIF)

S13 Fig Western blotting for muscular β-actin in Fig 6B. Representative image; the skeletal muscle was obtained from the STZ-induced diabetic mice following treatment with vehicle, BB, Metf, or Feno for 4 weeks.
(TIF)

S14 Fig Western blotting for hepatic phospho-Akt in Fig 6B. Representative image; the liver tissue was obtained from the STZ-induced diabetic mice following treatment with vehicle, BB, Metf, or Feno for 4 weeks.
(TIF)

S15 Fig Western blotting for hepatic total-Akt in Fig 6B. Representative image; the liver tissue was obtained from the STZ-induced diabetic mice following treatment with vehicle, BB, Metf, or Feno for 4 weeks.
(TIF)

S16 Fig Western blotting for hepatic β-actin in Fig 6B. Representative image; the liver tissue was obtained from the STZ-induced diabetic mice following treatment with vehicle, BB, Metf, or Feno for 4 weeks.
(TIF)

S17 Fig Western blotting for hepatic PPARα in Fig 7A. Representative image; the liver tissue was obtained from the STZ-induced diabetic mice following treatment with vehicle, BB, Metf, or Feno for 4 weeks.
(TIF)

S18 Fig Western blotting for hepatic FAS in Fig 7A. Representative image; the liver tissue was obtained from the STZ-induced diabetic mice following treatment with vehicle, BB, Metf, or Feno for 4 weeks.
(TIF)

S19 Fig Western blotting for hepatic PPARγ in Fig 7A. Representative image; the liver tissue was obtained from the STZ-induced diabetic mice following treatment with vehicle, BB, Metf, or Feno for 4 weeks.
(TIF)

S20 Fig Western blotting for hepatic β-actin in Fig 7A. Representative image; the liver tissue was obtained from the STZ-induced diabetic mice following treatment with vehicle, BB, Metf, or Feno for 4 weeks.
(TIF)

S21 Fig Western blotting for adipose PPARγ in Fig 7A. Representative image; the adipose tissue was obtained from the STZ-induced diabetic mice following treatment with vehicle, BB, Metf, or Feno for 4 weeks.
(TIF)

S22 Fig Western blotting for adipose FAS in Fig 7A. Representative image; the adipose tissue was obtained from the STZ-induced diabetic mice following treatment with vehicle, BB, Metf, or Feno for 4 weeks.
(TIF)

S23 Fig Western blotting for adipose β-actin in Fig 7A. Representative image; the adipose tissue was adapted from the STZ-induced diabetic mice following treatment with vehicle, BB, Metf, or Feno for 4 weeks.
(TIF)

## Abbreviations

11β-HSD1: 11beta hydroxysteroid dehydrogenase
AMPK: AMP-activated protein kinase
aP2: Adipocyte fatty acid binding protein 2
BAT: Brown adipose tissue
CON: Control
CPT1a: carnitine palmitoyl transferase Ia
EWAT: Epididymal white adipose tissue
FAS: Fatty acid synthase
Feno: Fenofibrate
FFA: Free fatty acid
GAPDH: Glyceraldehyde-3-phosphate dehydrogenase
G6 Pase: Glucose-6-phosphatase
GLUT4: Glucose transporter 4
Metf: Metformin
PEPCK: phosphoenolpyruvate carboxykinase
PPAR: Peroxisome proliferator-activated receptor
RT-PCR: Reverse transcription-polymerase chain reaction
RWAT: Retroperitoneal white adipose tissue
SREBP: Sterol regulatory element binding protein
TC: Total cholesterol
TG: Triglyceride
WAT: White adipose tissue
