# Supplementary material for: (−)-Epicatechin-3-O-β-D-allopyranoside from Davallia formosana prevents diabetes and dyslipidemia in streptozotocin-induced diabetic mice
Source: PLoS One. 2017 Mar 23;12(3):e0173984. doi: 10.1371/journal.pone.0173984 (PMC5363832; doi:10.1371/journal.pone.0173984)
Supplement: S1 Animal Supplement — (PDF) [file pone.0173984.s001.pdf]

S1 Animal supplement. Animal Ethics. This described the early euthanasia/ humane endpoints for animals those became severely ill to reduce pain to the minimum degree during the experiments, the clinical signs used to determine when to euthanize the animals including the following issues: 1. the decreased body weights by loss of 25% original body weights or animals display cachexia or consumptive syndromes; for the non-grow-stage animals: the decreased body weights depend on the body weights of animals entering the room or the body weights of mean aged animals; the grow-stage animals may be not display decreased body weights, and if they display not normally increasing body weight to be classified into the decreased body weights; 2. for rodents: the decreased food intake within 3 days (food intake is below 50% of normal ingestions); 3. weak or dying status: animals (without any anesthesia or sedatives) could not stand up; 4. infection: no matter what obviously evident by the reason of body temperature caused high WBC levels and diagnose is due to infection; following treatment with antibiotics, animals display ineffective and systemic uncomfortable syndrome; 5. organs of animals with severe loss of functions and with clinical symptoms; following treatment displayed ineffective and veterinarians made diagnoses to be prognosis not good; 6. tumor: growth weights above 10% body weights; 7. Existed pain and it could not be controlled following treatment with analgesics.
