# Supplementary figures and images for: (−)-Epicatechin-3-O-β-D-allopyranoside from Davallia formosana prevents diabetes and dyslipidemia in streptozotocin-induced diabetic mice
Source: PLoS One. 2017 Mar 23;12(3):e0173984. doi: 10.1371/journal.pone.0173984 (PMC5363832; doi:10.1371/journal.pone.0173984)

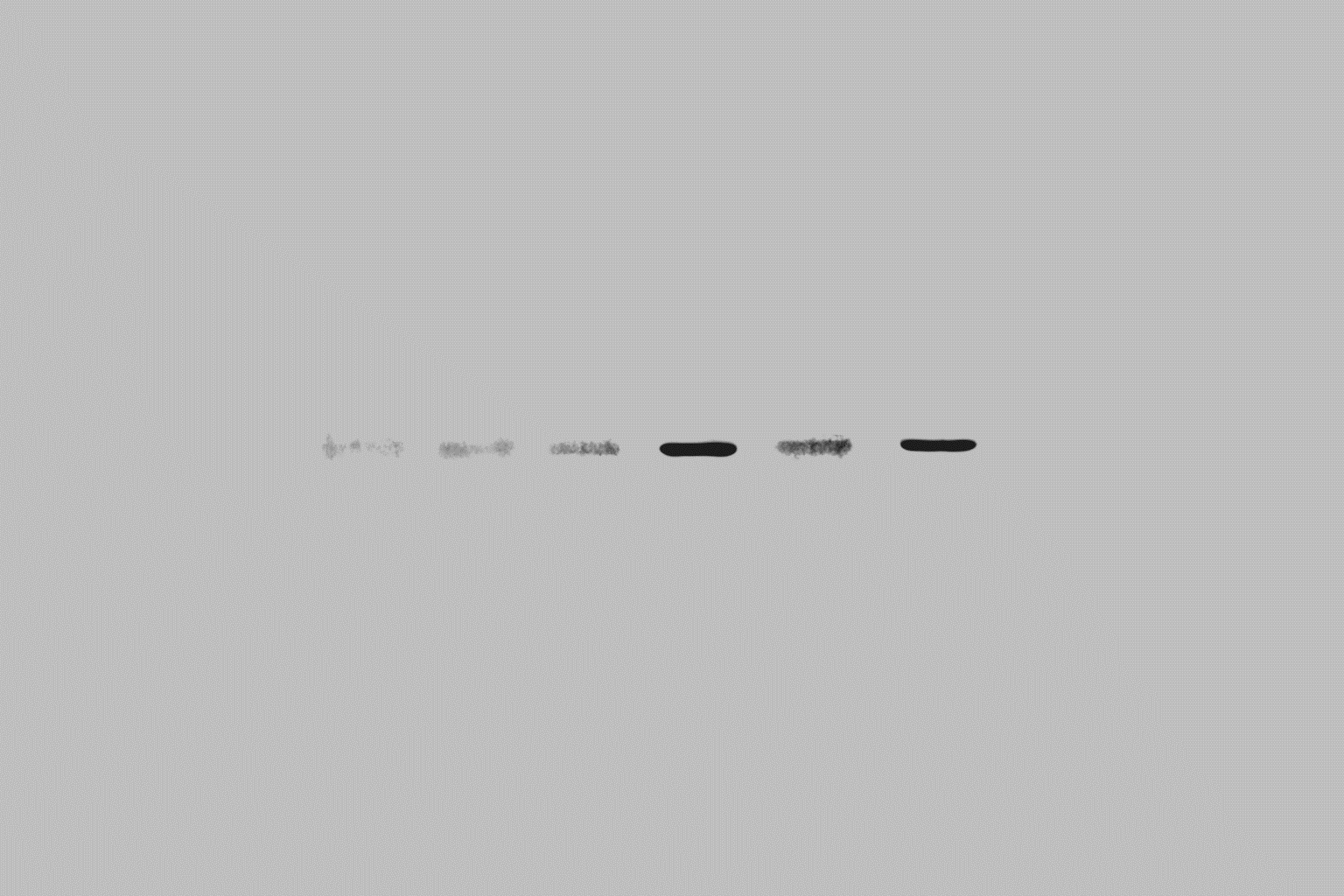

Supplement: S1 Fig — Representative image; Akt phosphorylation was determined from C2C12 cells, and treated with 40 μg/ mL of BB for the indicated period of time (5–60 min). (TIF) [file pone.0173984.s002.tif]

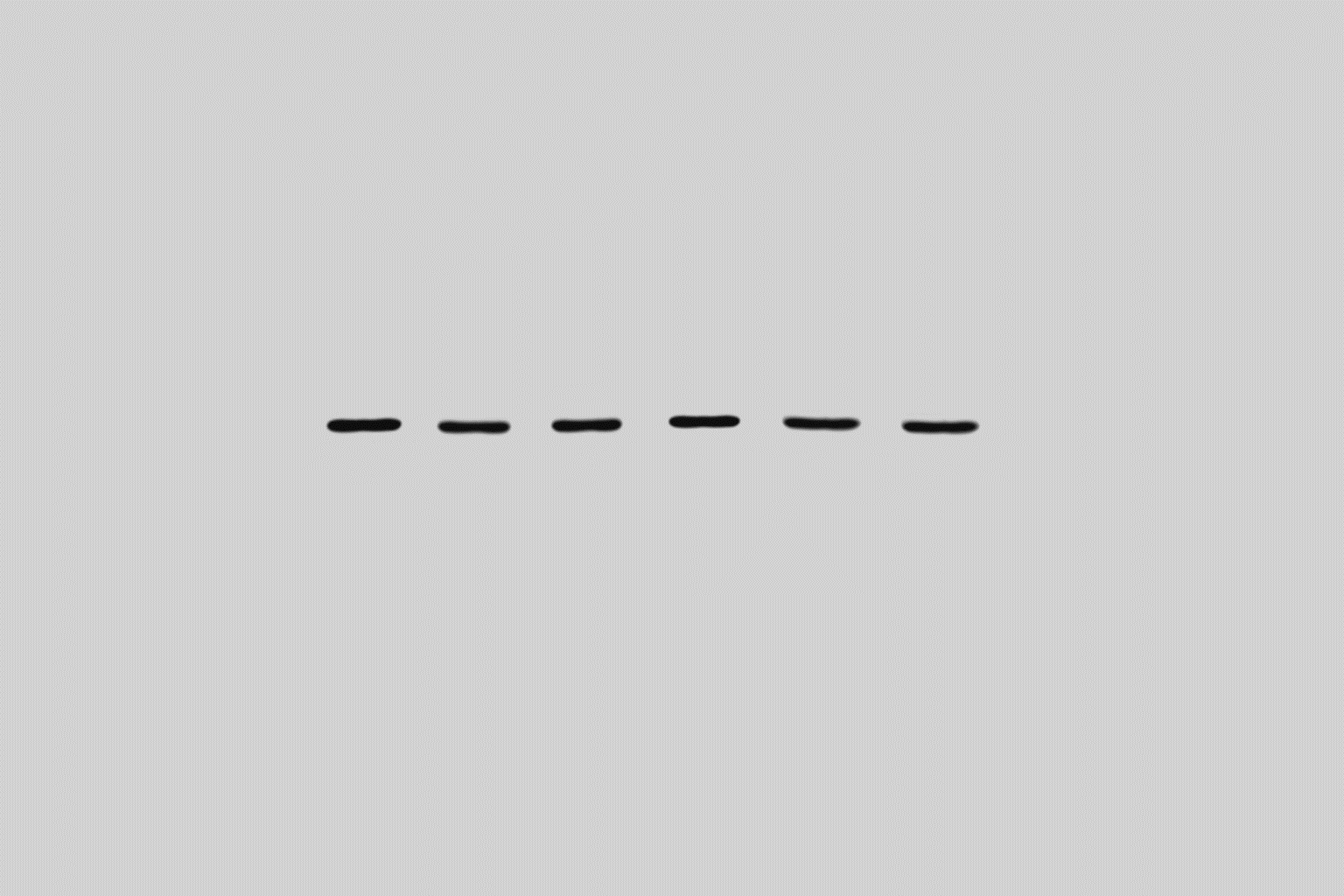

Supplement: S2 Fig — Representative image; total-Akt was determined from C2C12 cells, and treated with 40 μg/ mL of BB for the indicated period of time (5–60 min). (TIF) [file pone.0173984.s003.tif]

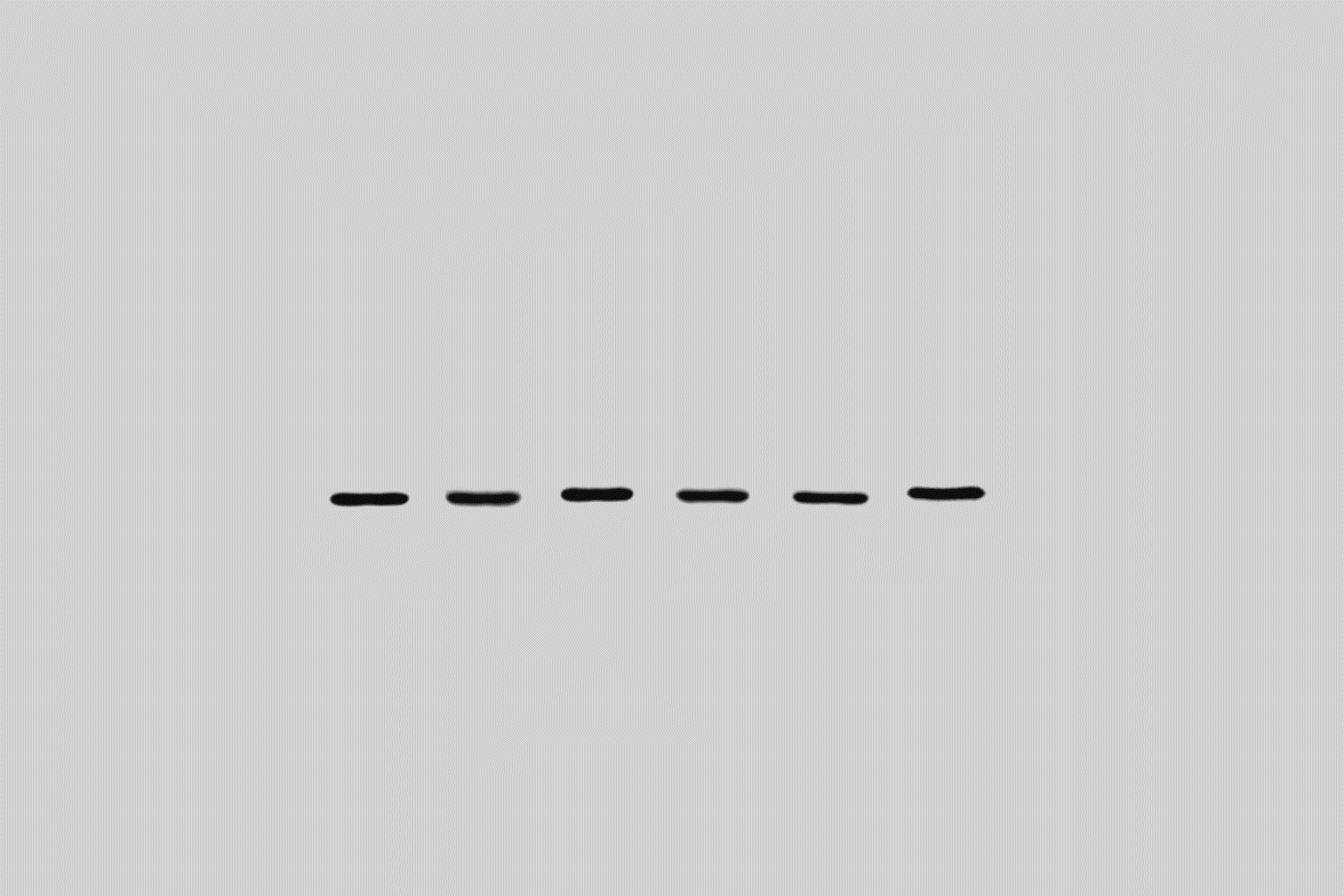

Supplement: S3 Fig — Representative image; β-actin was determined from C2C12 cells, and treated with 40 μg/ mL of BB for the indicated period of time (5–60 min). (TIF) [file pone.0173984.s004.tif]

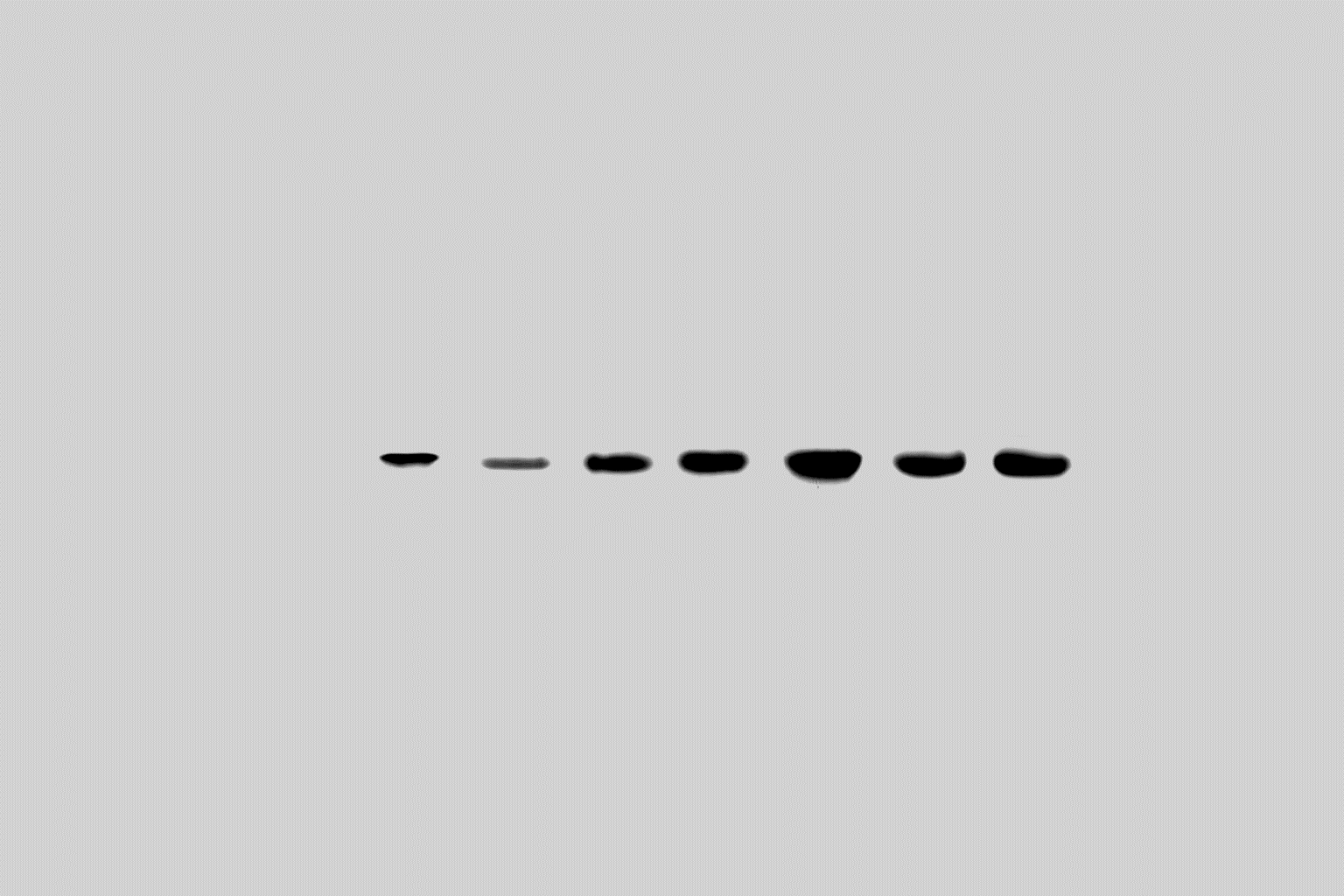

Supplement: S4 Fig — Representative image; the skeletal muscle was obtained from the STZ-induced diabetic mice following treatment with vehicle, BB, Metf, or Feno for 4 weeks. (TIF) [file pone.0173984.s005.tif]

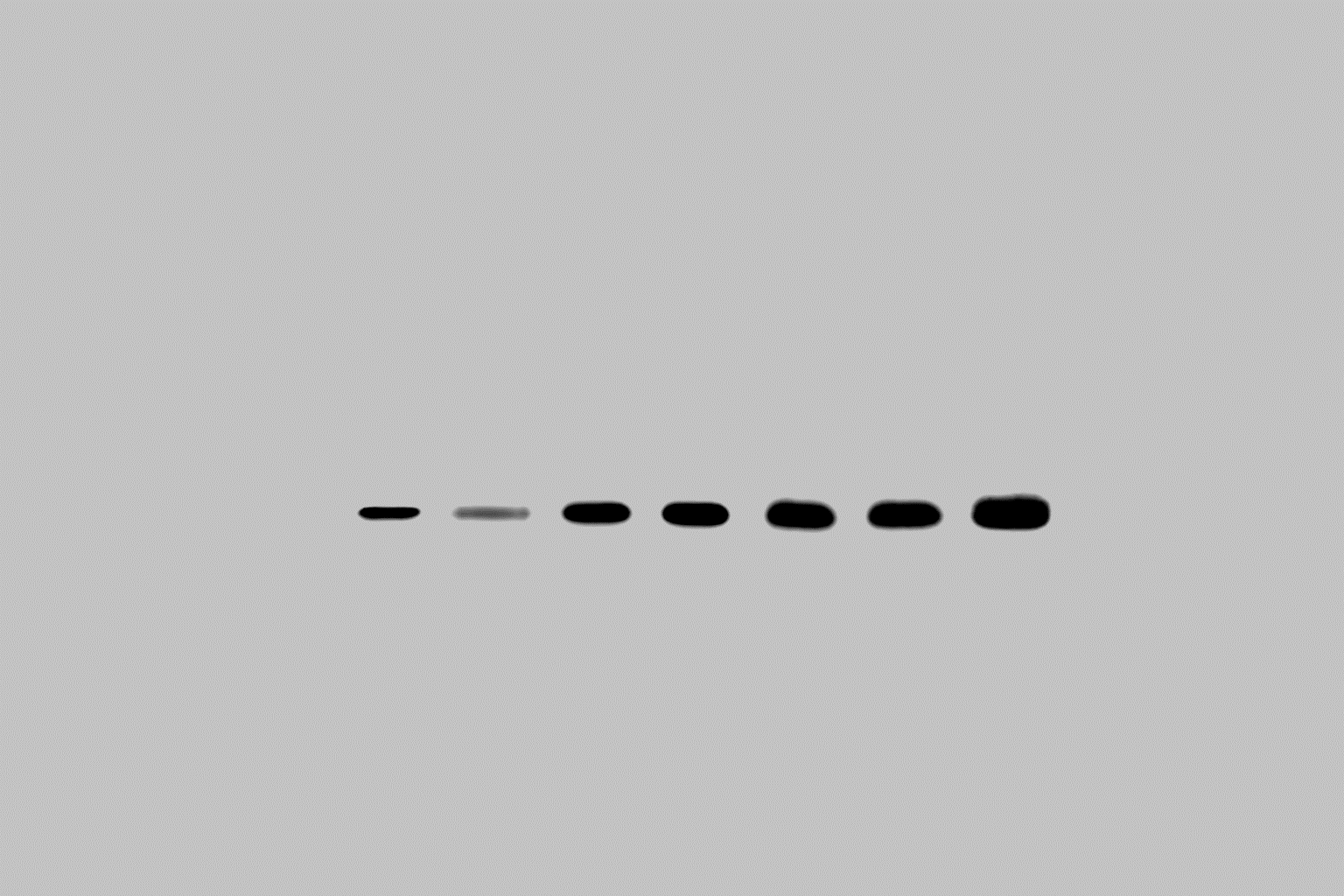

Supplement: S5 Fig — Representative image; the skeletal muscle was obtained from the STZ-induced diabetic mice following treatment with vehicle, BB, Metf, or Feno for 4 weeks. (TIF) [file pone.0173984.s006.tif]

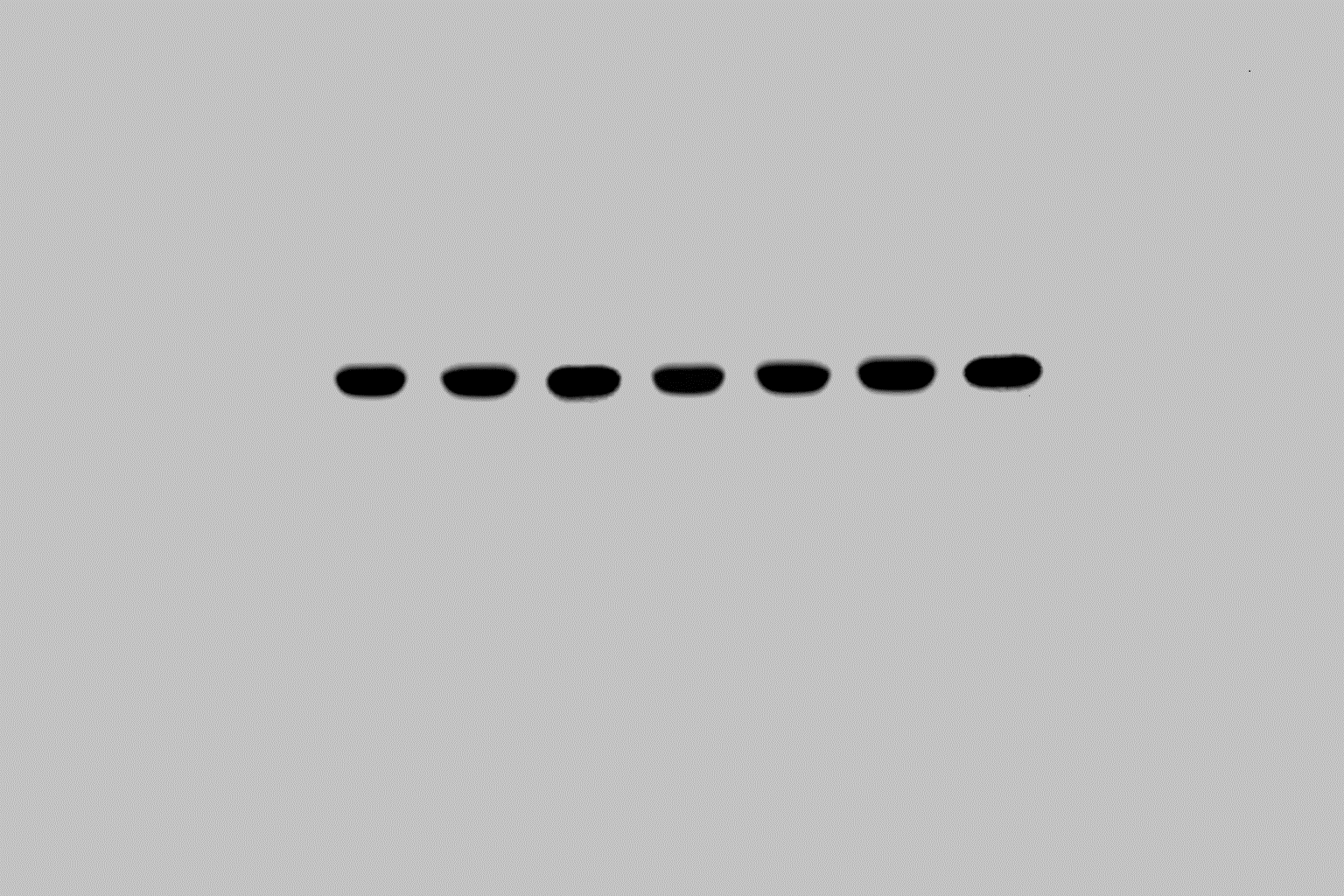

Supplement: S6 Fig — Representative image; the skeletal muscle was obtained from the STZ-induced diabetic mice following treatment with vehicle, BB, Metf, or Feno for 4 weeks. (TIF) [file pone.0173984.s007.tif]

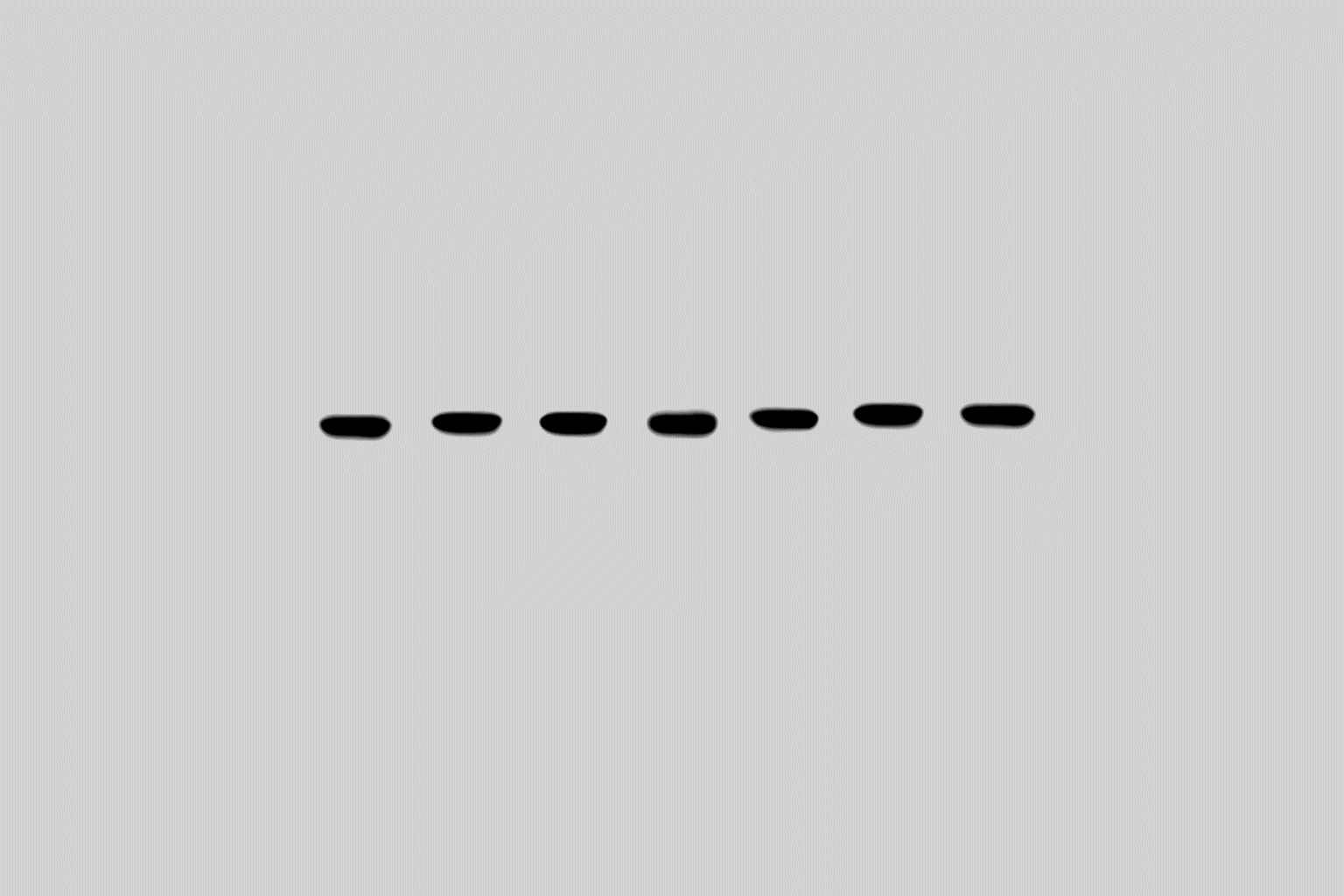

Supplement: S7 Fig — Representative image; the skeletal muscle was obtained from the STZ-induced diabetic mice following treatment with vehicle, BB, Metf, or Feno for 4 weeks. (TIF) [file pone.0173984.s008.tif]

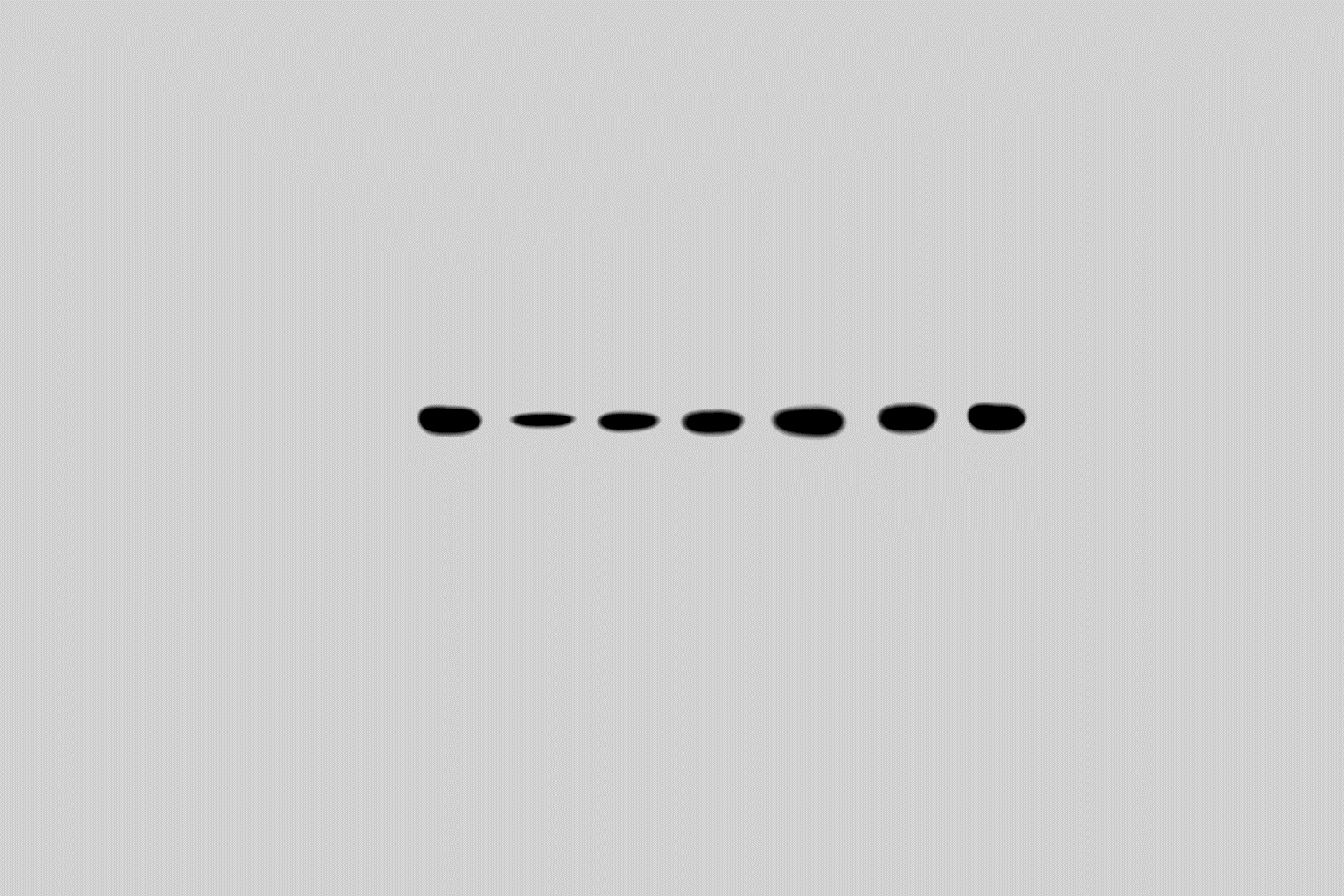

Supplement: S8 Fig — Representative image; the liver tissue was obtained from the STZ-induced diabetic mice following treatment with vehicle, BB, Metf, or Feno for 4 weeks. (TIF) [file pone.0173984.s009.tif]

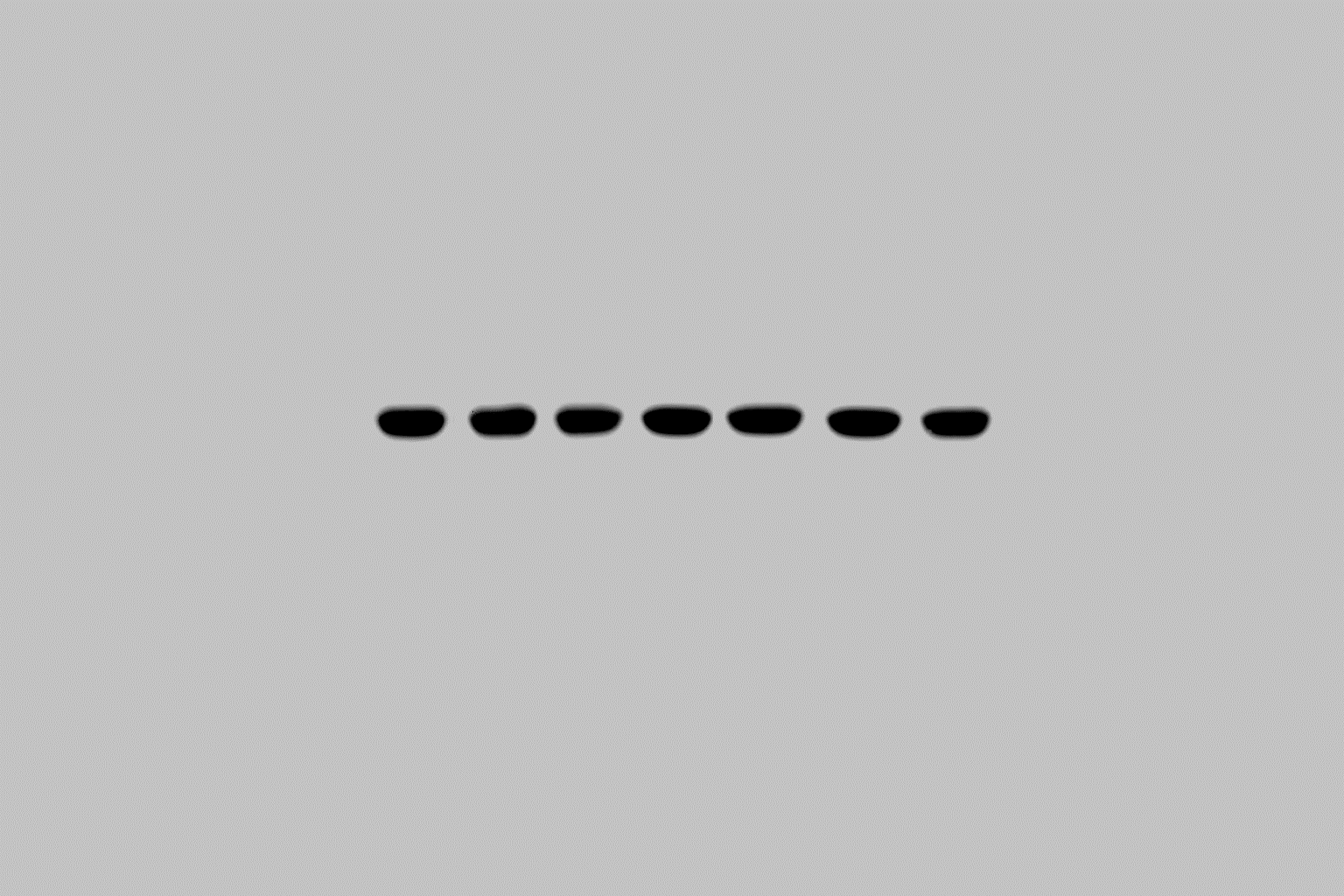

Supplement: S9 Fig — Representative image; the liver tissue was obtained from the STZ-induced diabetic mice following treatment with vehicle, BB, Metf, or Feno for 4 weeks. (TIF) [file pone.0173984.s010.tif]

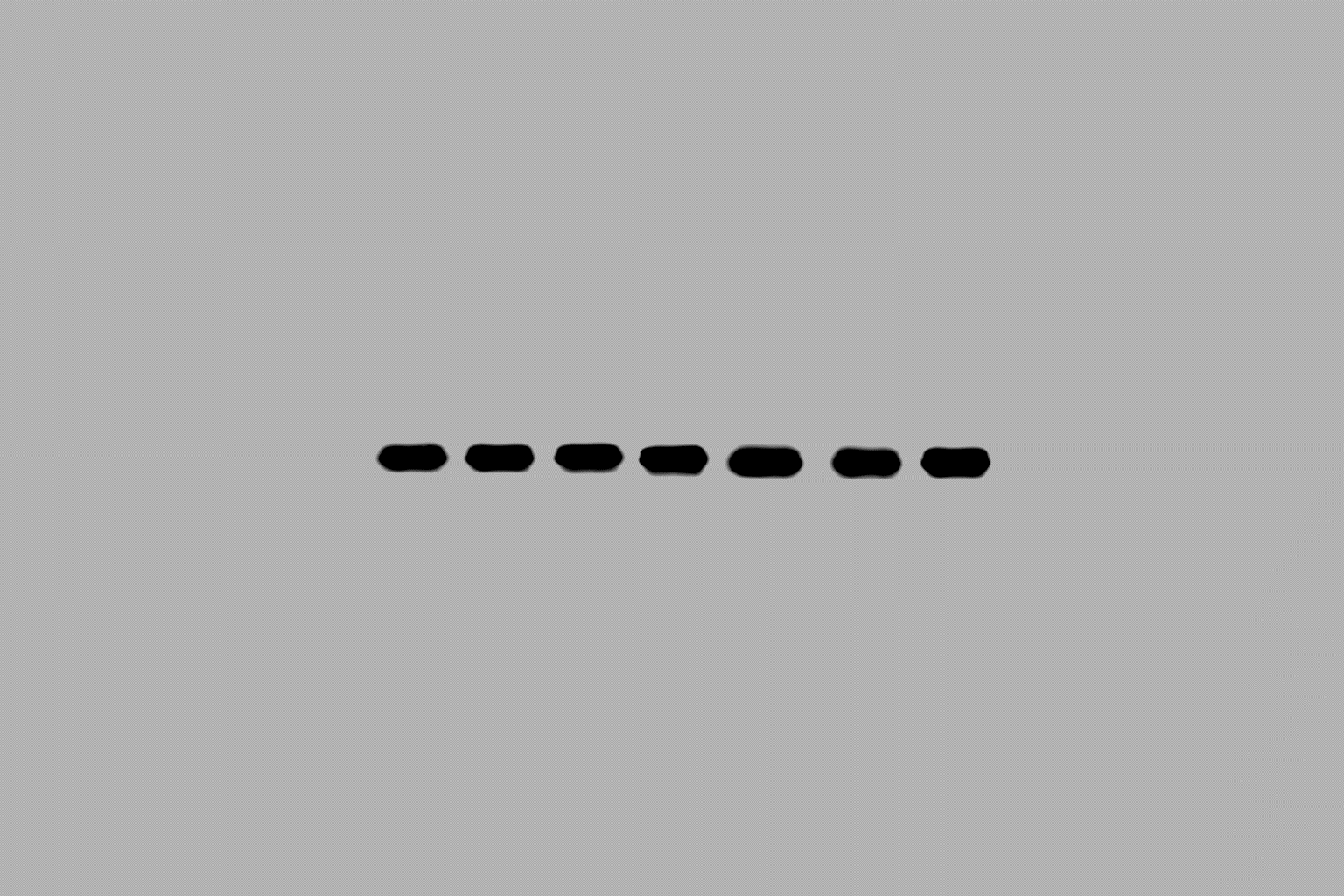

Supplement: S10 Fig — Representative image; the liver tissue was obtained from the STZ-induced diabetic mice following treatment with vehicle, BB, Metf, or Feno for 4 weeks. (TIF) [file pone.0173984.s011.tif]

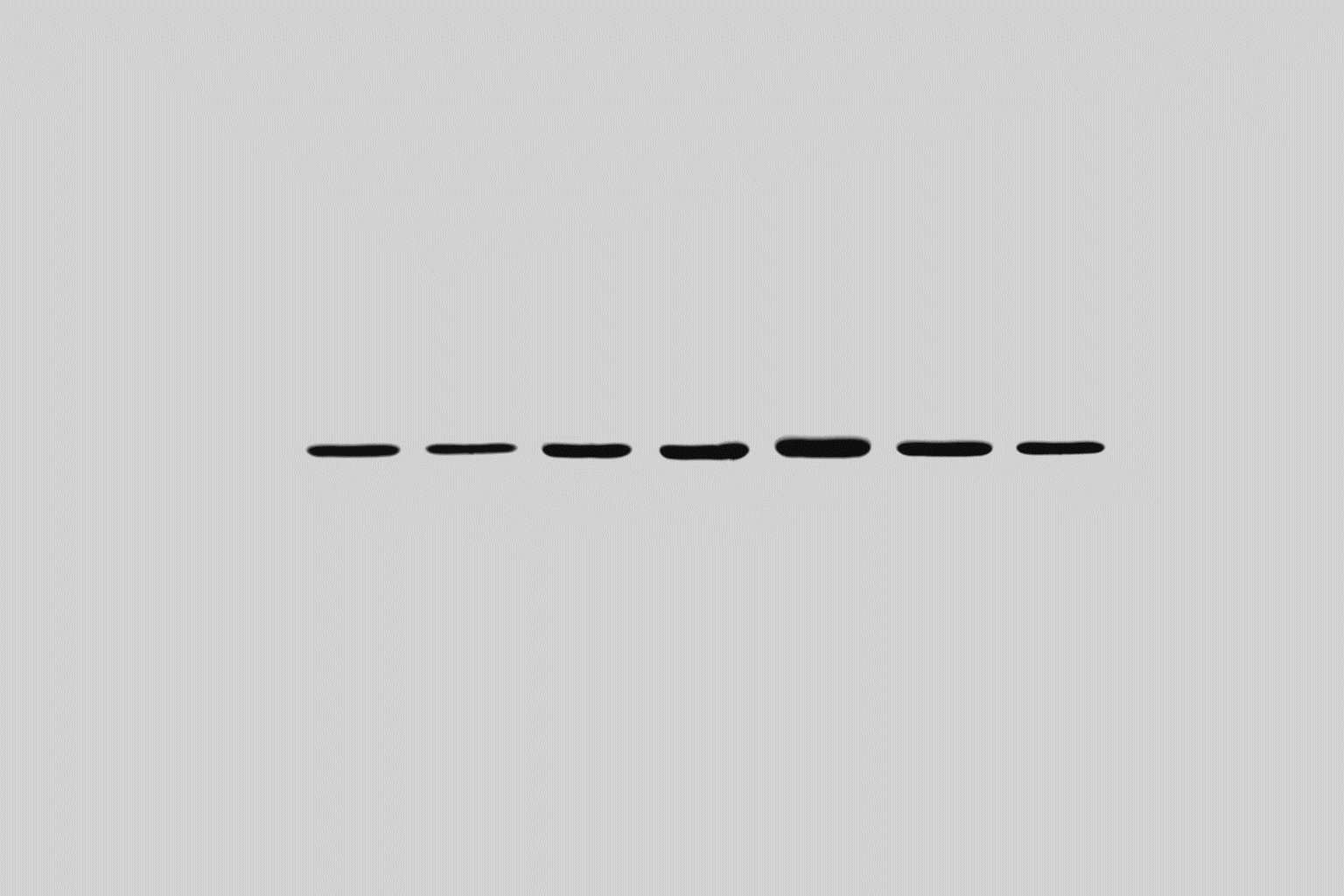

Supplement: S11 Fig — Representative image; the skeletal muscle was obtained from the STZ-induced diabetic mice following treatment with vehicle, BB, Metf, or Feno for 4 weeks. (TIF) [file pone.0173984.s012.tif]

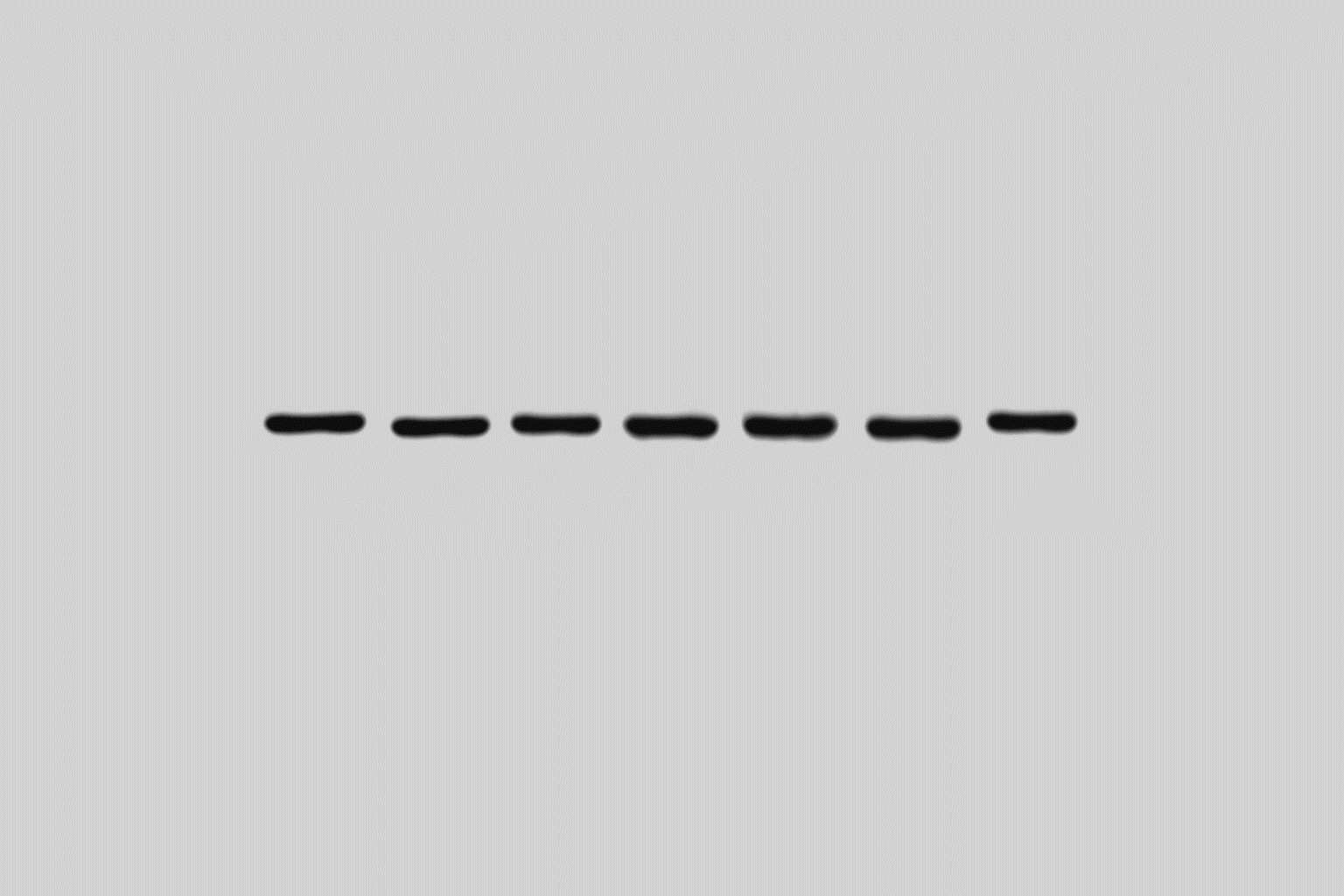

Supplement: S12 Fig — Representative image; the skeletal muscle was obtained from the STZ-induced diabetic mice following treatment with vehicle, BB, Metf, or Feno for 4 weeks. (TIF) [file pone.0173984.s013.tif]

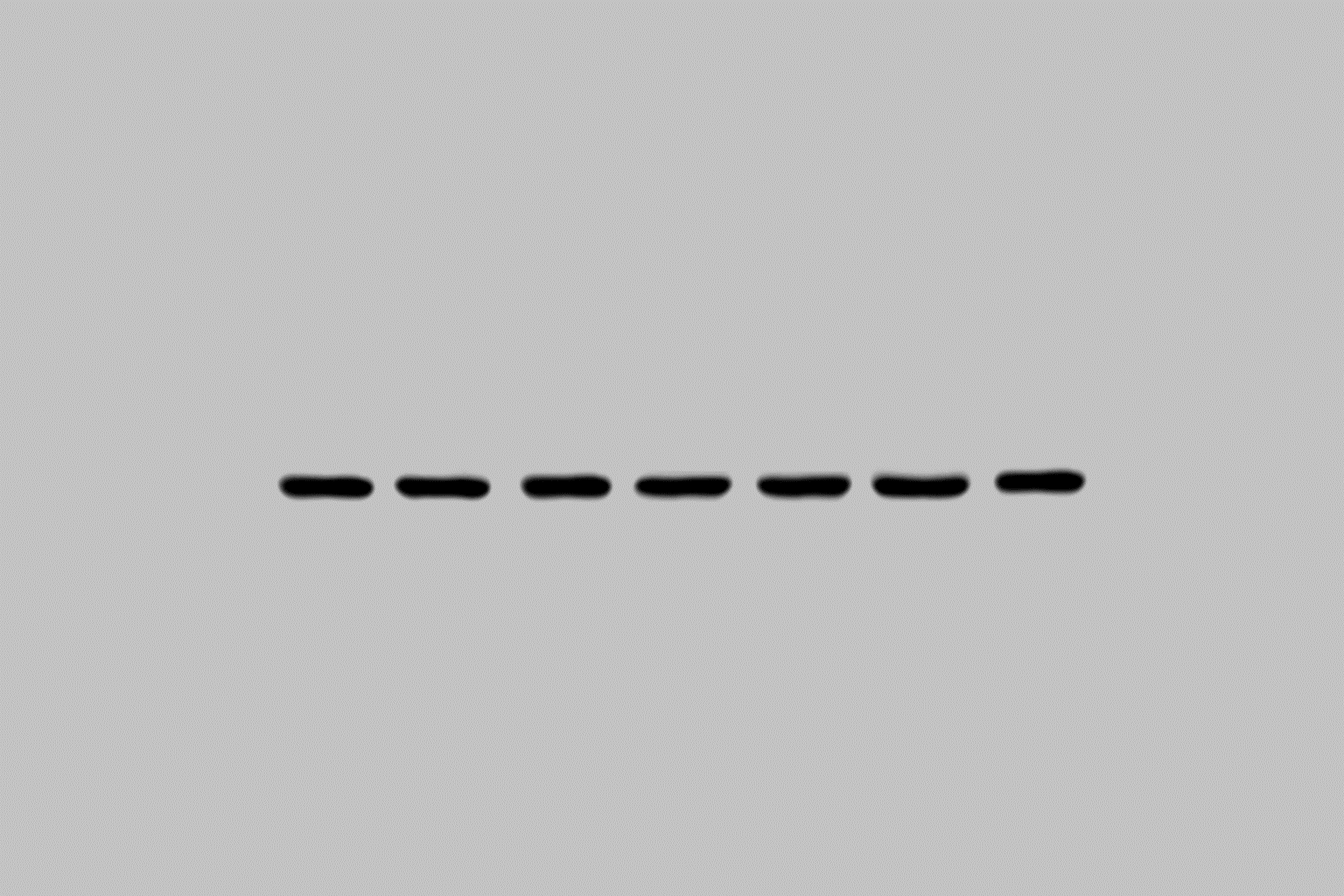

Supplement: S13 Fig — Representative image; the skeletal muscle was obtained from the STZ-induced diabetic mice following treatment with vehicle, BB, Metf, or Feno for 4 weeks. (TIF) [file pone.0173984.s014.tif]

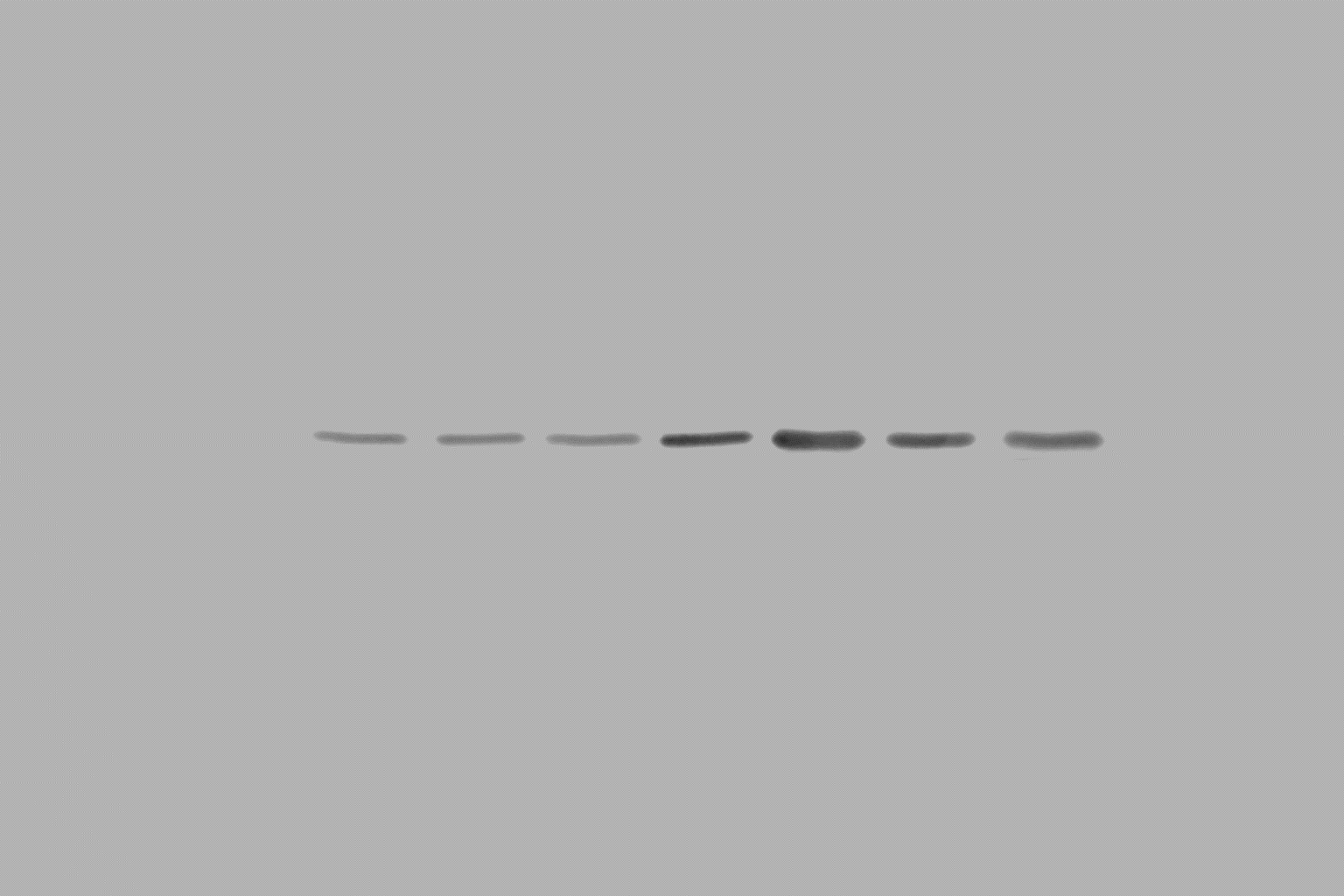

Supplement: S14 Fig — Representative image; the liver tissue was obtained from the STZ-induced diabetic mice following treatment with vehicle, BB, Metf, or Feno for 4 weeks. (TIF) [file pone.0173984.s015.tif]

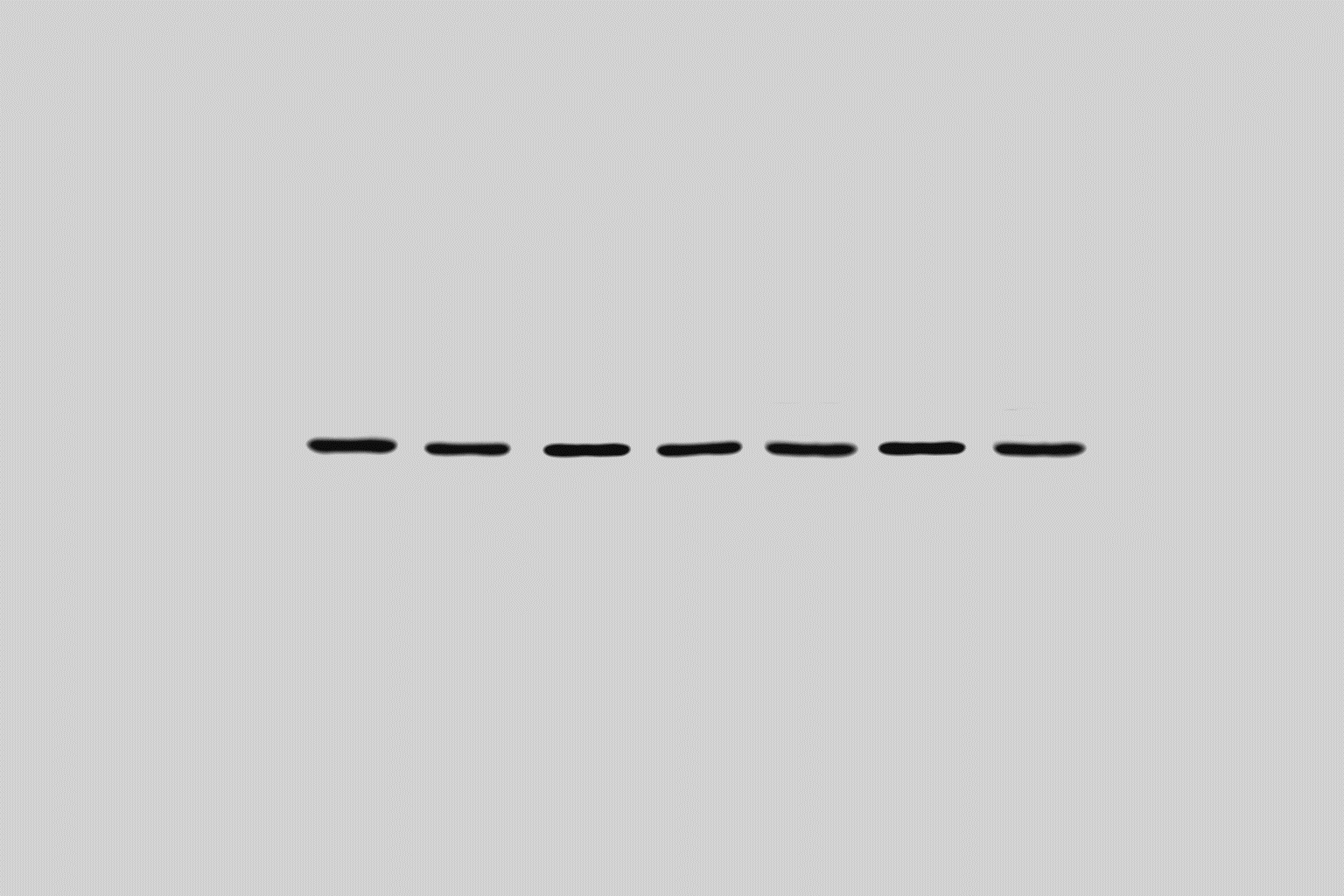

Supplement: S15 Fig — Representative image; the liver tissue was obtained from the STZ-induced diabetic mice following treatment with vehicle, BB, Metf, or Feno for 4 weeks. (TIF) [file pone.0173984.s016.tif]

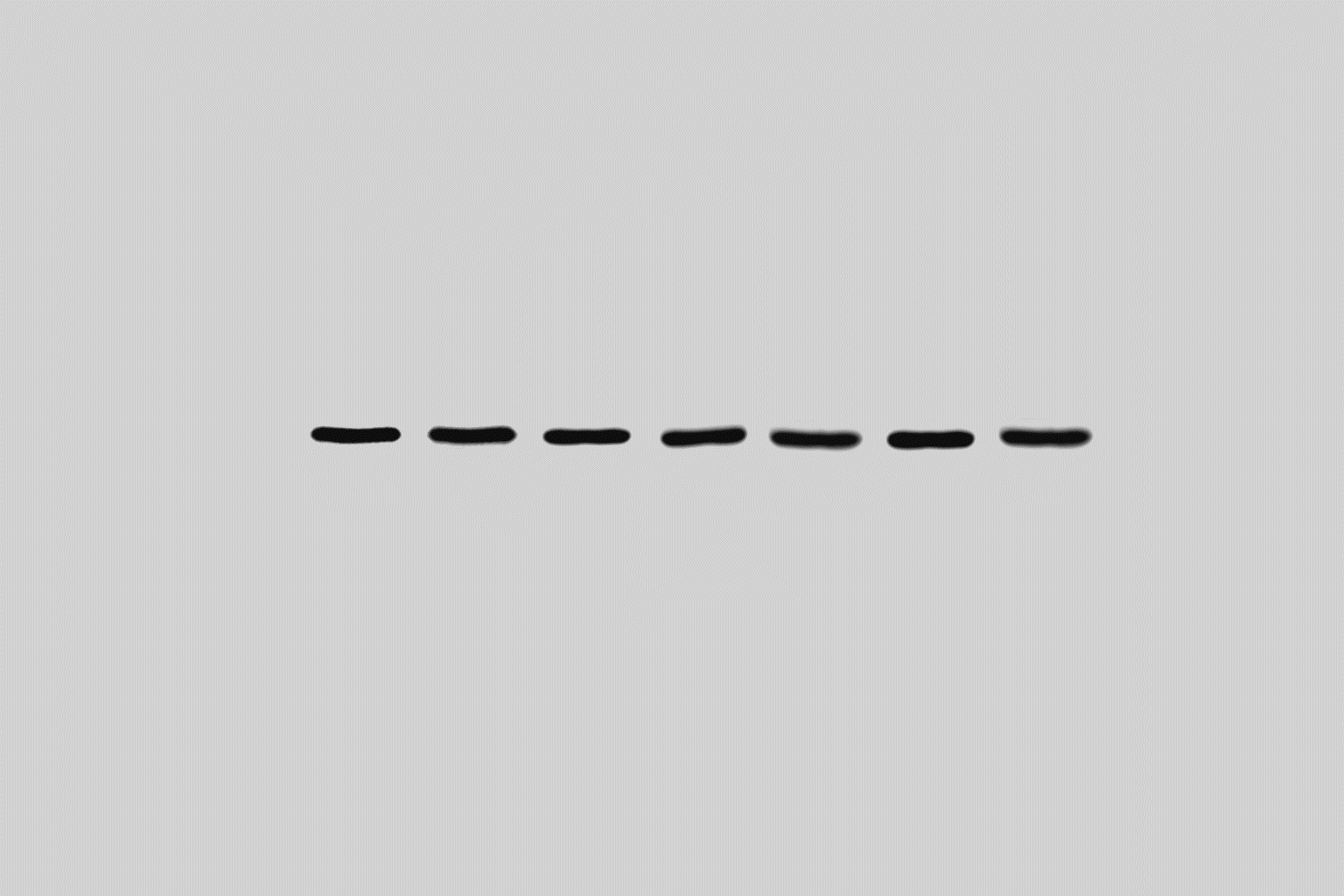

Supplement: S16 Fig — Representative image; the liver tissue was obtained from the STZ-induced diabetic mice following treatment with vehicle, BB, Metf, or Feno for 4 weeks. (TIF) [file pone.0173984.s017.tif]

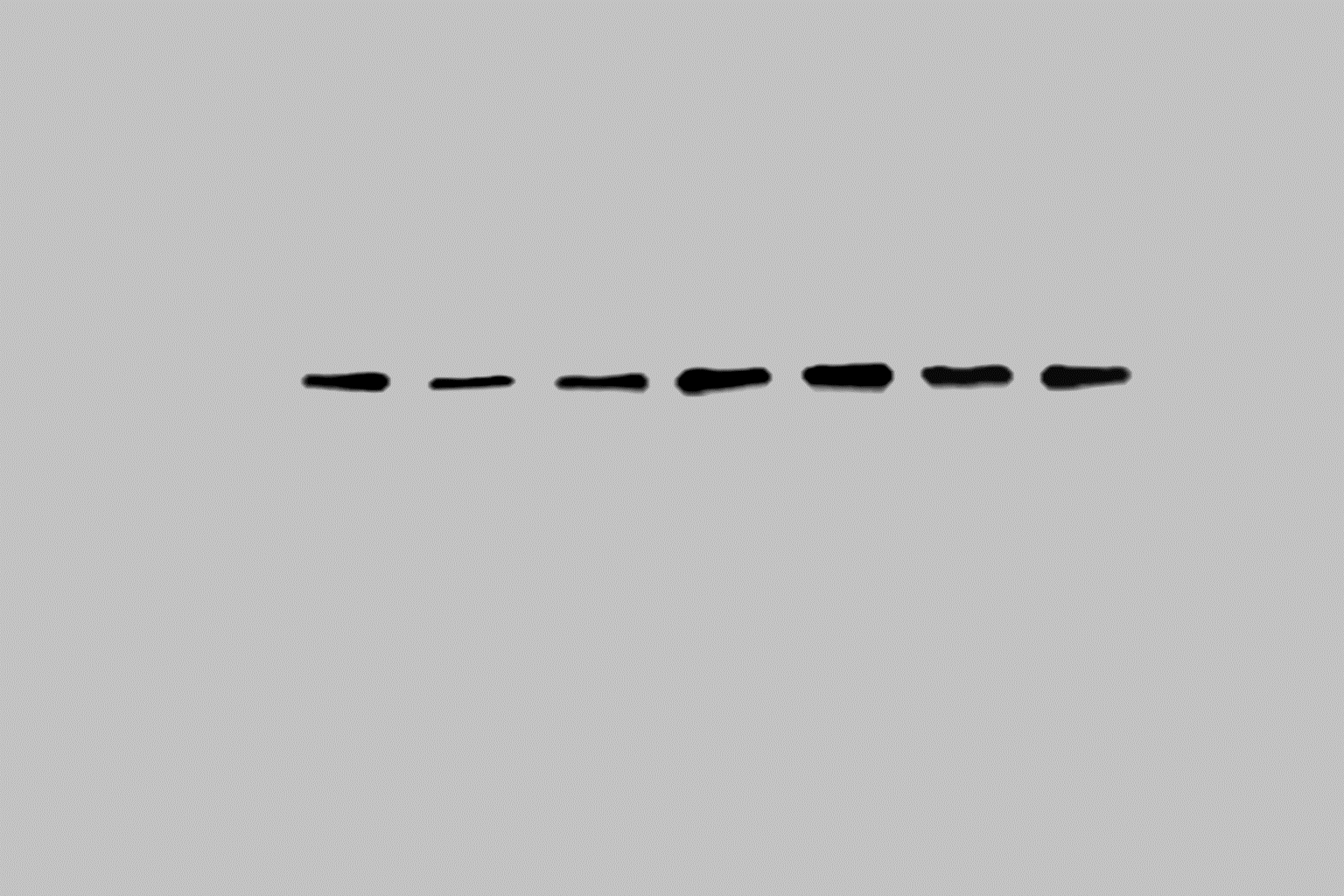

Supplement: S17 Fig — Representative image; the liver tissue was obtained from the STZ-induced diabetic mice following treatment with vehicle, BB, Metf, or Feno for 4 weeks. (TIF) [file pone.0173984.s018.tif]

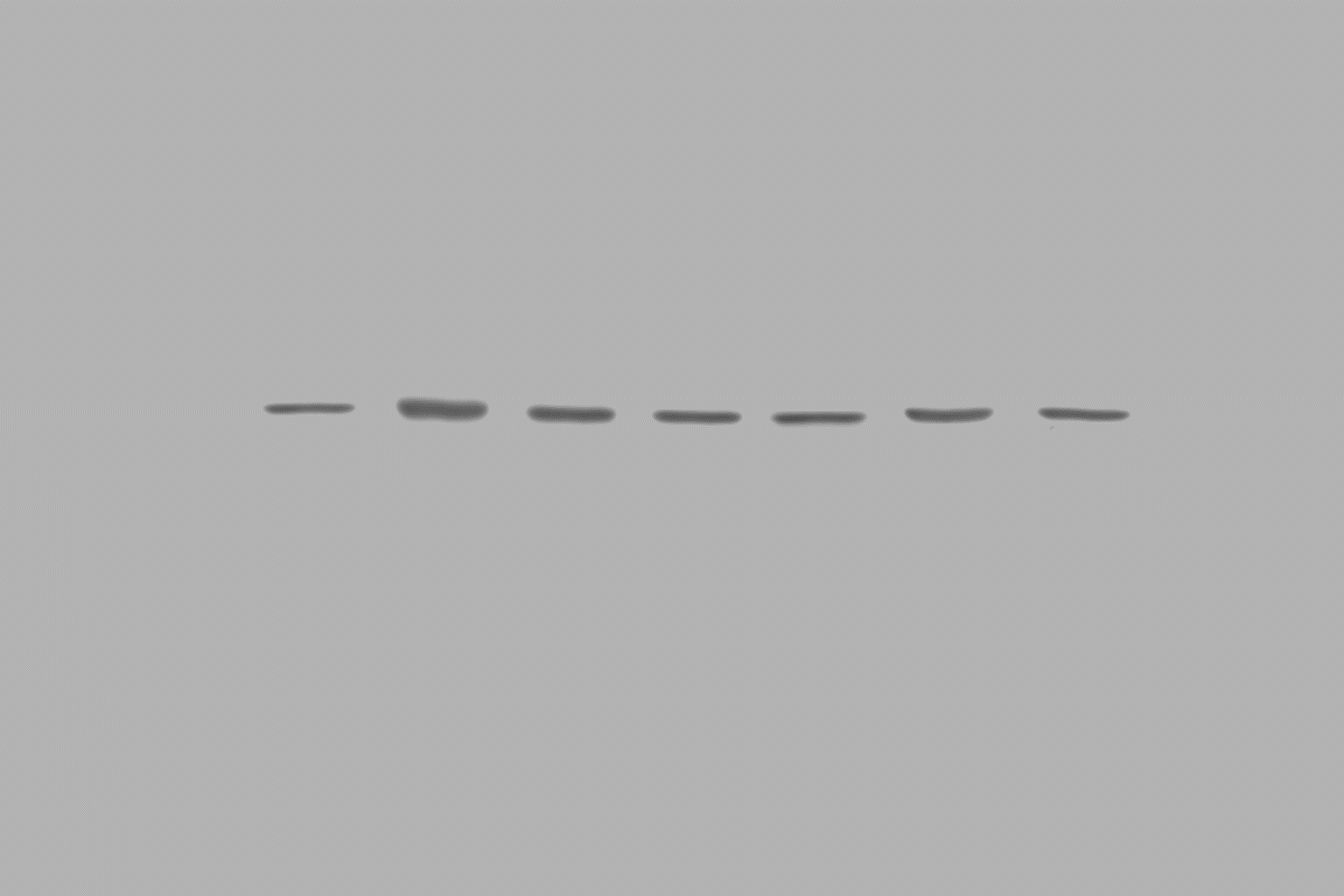

Supplement: S18 Fig — Representative image; the liver tissue was obtained from the STZ-induced diabetic mice following treatment with vehicle, BB, Metf, or Feno for 4 weeks. (TIF) [file pone.0173984.s019.tif]

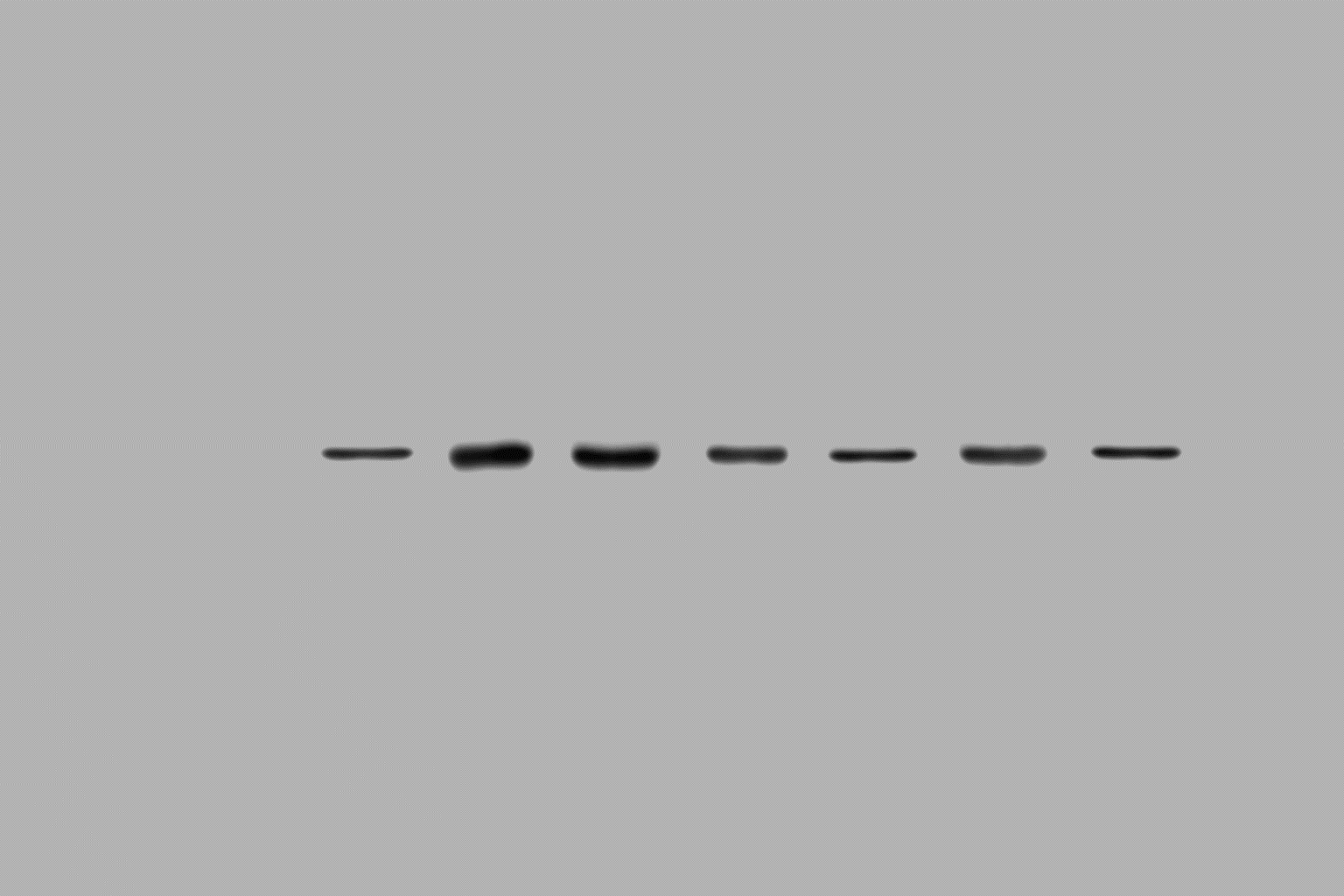

Supplement: S19 Fig — Representative image; the liver tissue was obtained from the STZ-induced diabetic mice following treatment with vehicle, BB, Metf, or Feno for 4 weeks. (TIF) [file pone.0173984.s020.tif]

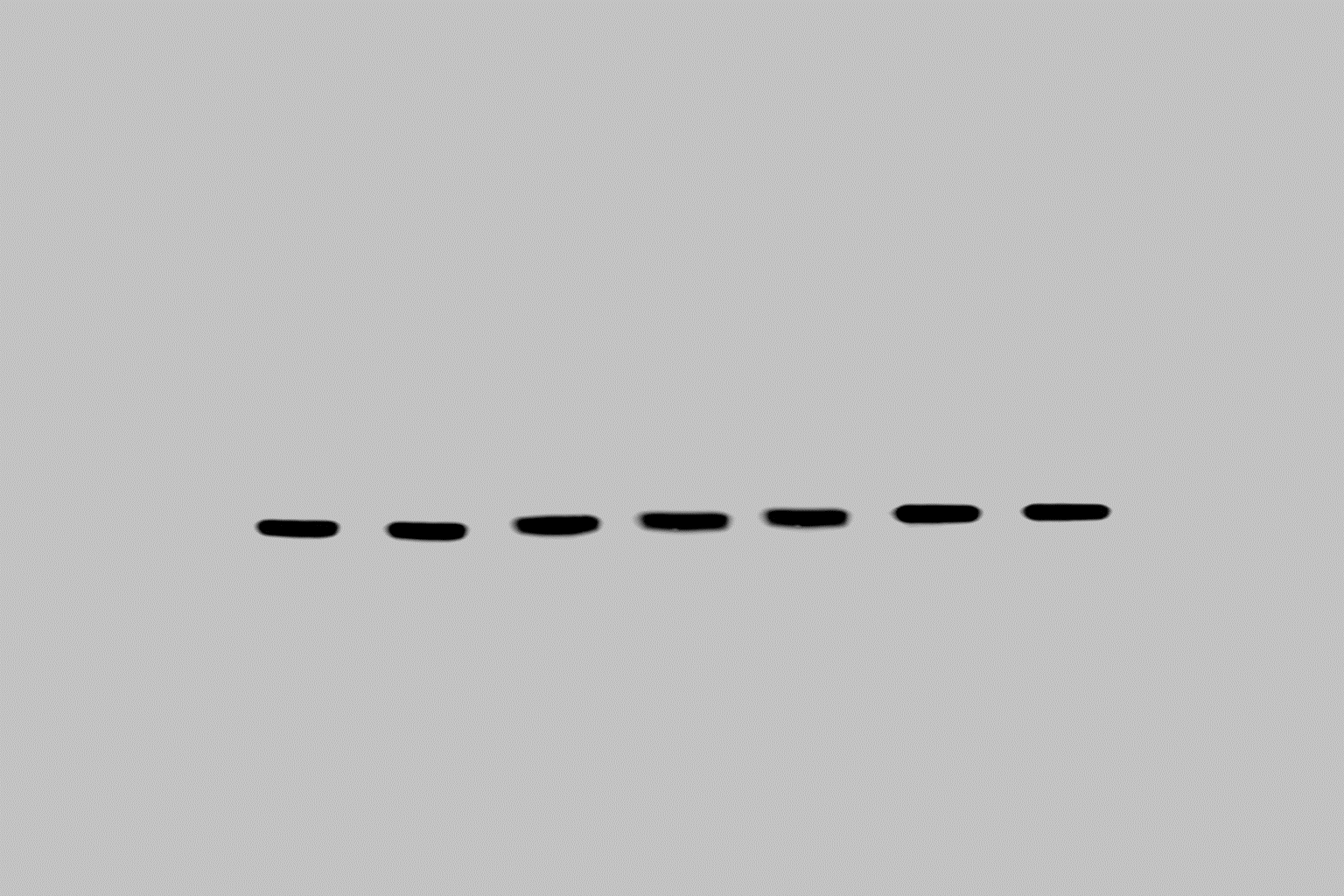

Supplement: S20 Fig — Representative image; the liver tissue was obtained from the STZ-induced diabetic mice following treatment with vehicle, BB, Metf, or Feno for 4 weeks. (TIF) [file pone.0173984.s021.tif]

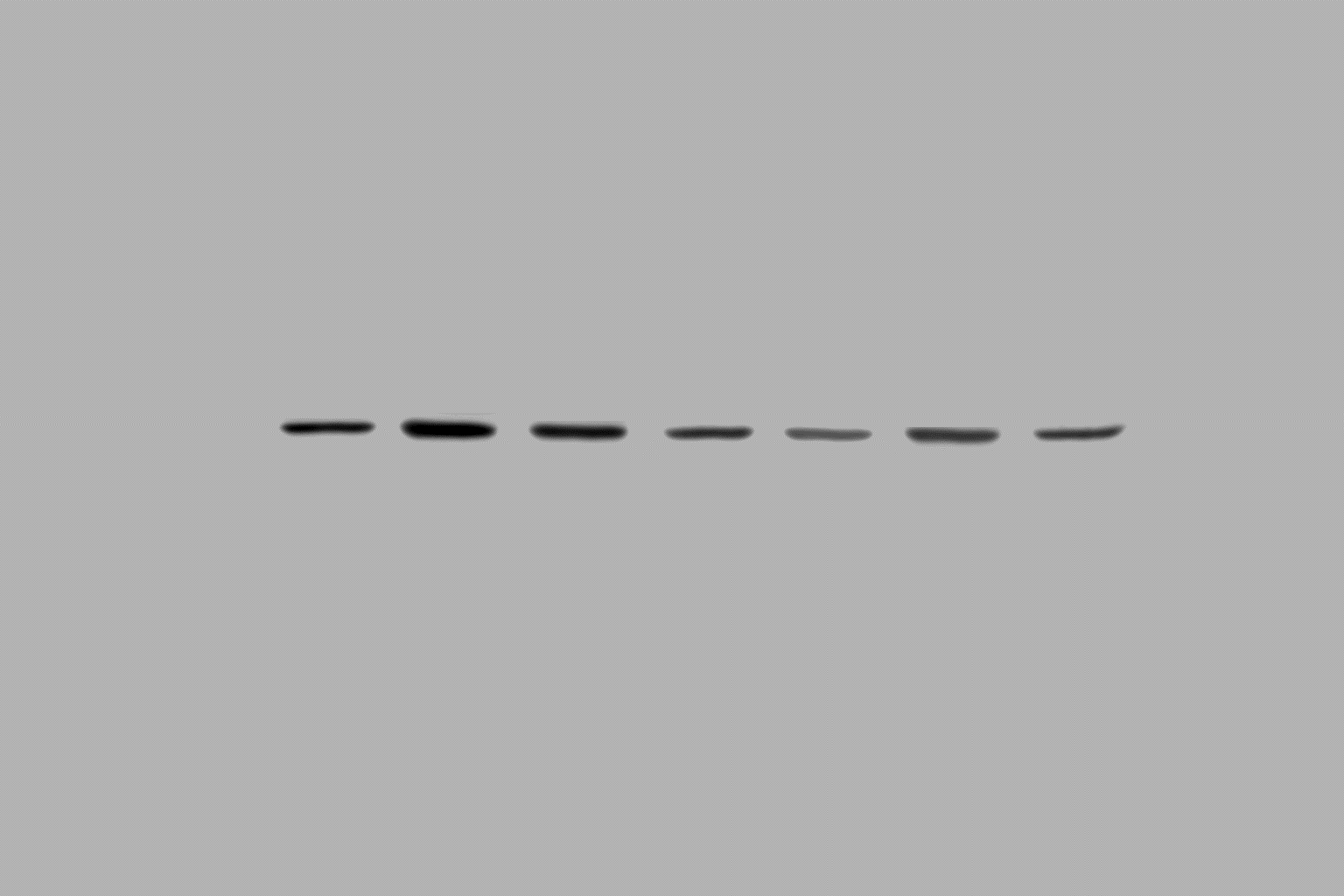

Supplement: S21 Fig — Representative image; the adipose tissue was obtained from the STZ-induced diabetic mice following treatment with vehicle, BB, Metf, or Feno for 4 weeks. (TIF) [file pone.0173984.s022.tif]

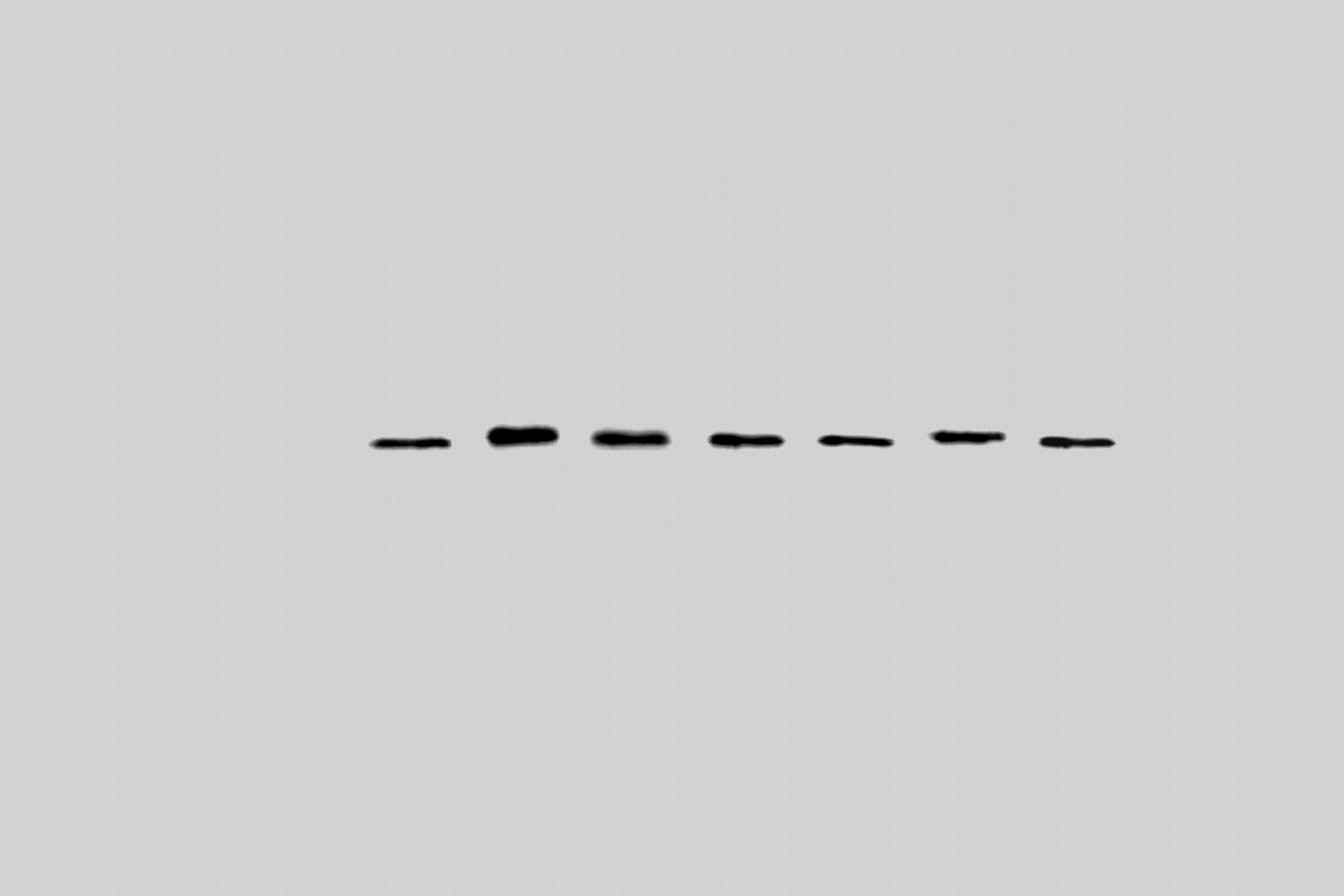

Supplement: S22 Fig — Representative image; the adipose tissue was obtained from the STZ-induced diabetic mice following treatment with vehicle, BB, Metf, or Feno for 4 weeks. (TIF) [file pone.0173984.s023.tif]

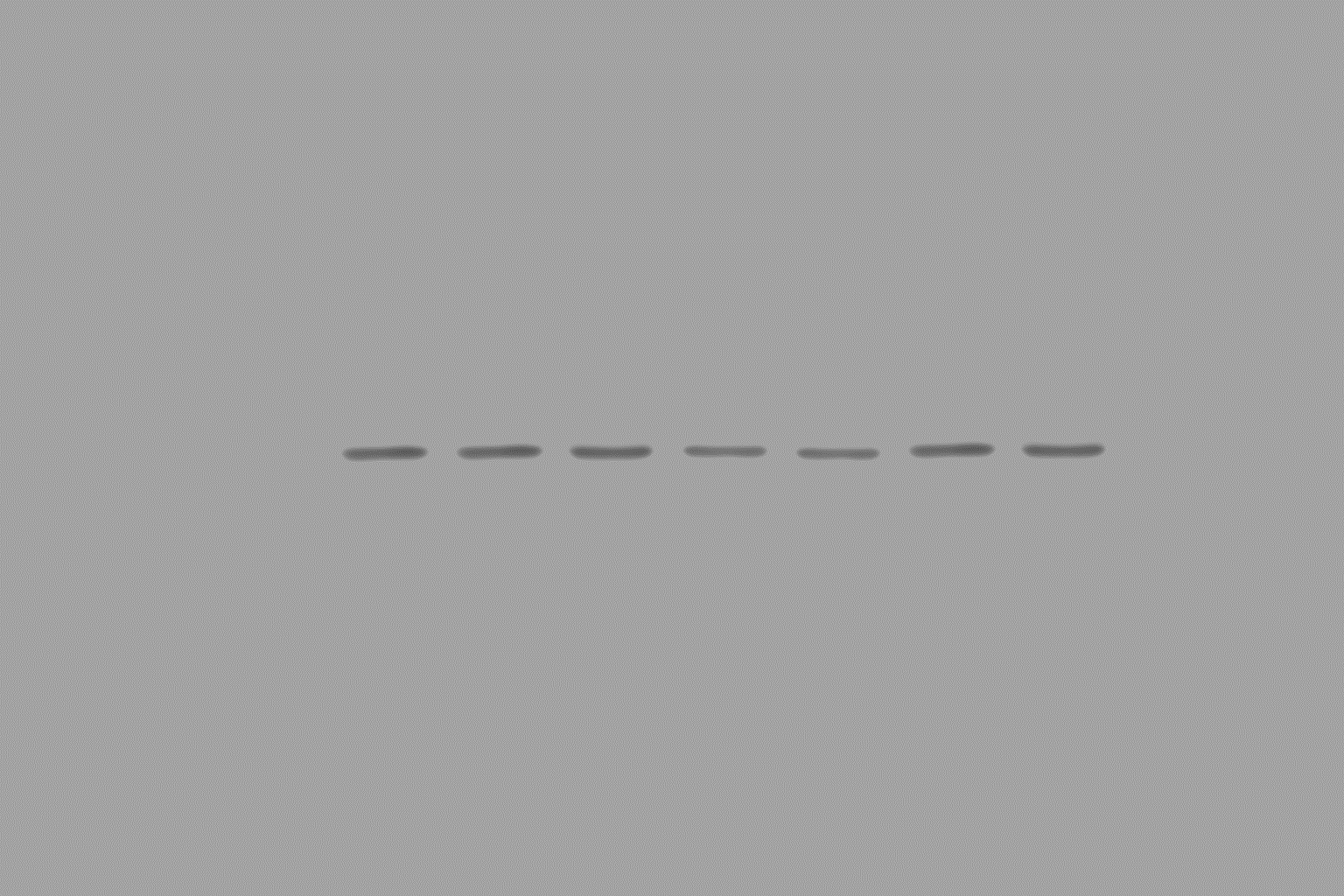

Supplement: S23 Fig — Representative image; the adipose tissue was adapted from the STZ-induced diabetic mice following treatment with vehicle, BB, Metf, or Feno for 4 weeks. (TIF) [file pone.0173984.s024.tif]
